# Integrated MALDI-TOF MS, Microbiological, Physicochemical and Sensory Assessment of Spoilage in Vacuum-Packaged Chicken Breast During Refrigerated Storage

**DOI:** 10.3390/foods15122162

**Published:** 2026-06-15

**Authors:** Nursel Söylemez Milli

**Affiliations:** Scientific, Industrial and Technological Application and Research Centre, Bolu Abant İzzet Baysal University, 14030 Bolu, Türkiye; nurselsoylemez@ibu.edu.tr

**Keywords:** vacuum packaging, chicken breast, MALDI-TOF MS, physicochemical properties, microbial succession, sensory evaluation, shelf life, spoilage-associated taxa

## Abstract

Spoilage in vacuum-packaged chicken breast is driven by coupled microbial succession and physicochemical changes that cannot be adequately described by a single indicator. In this study, MALDI-TOF MS-based species-level identification of culturable isolates was integrated with microbiological counts (total viable count, lactic acid bacteria, yeasts and molds, and Enterobacteriaceae), physicochemical parameters (pH, water activity, CIE $L^{*}a^{*}b^{*}$, and total volatile basic nitrogen (TVB-N)), and sensory evaluation of odor, appearance/color, surface texture/slime and overall acceptability (trained panel, n=8) during 15 days of storage at 4 °C. Associations among variables were assessed using Spearman correlation analysis. MALDI-TOF MS identified 625 isolates belonging to 67 species across 19 families. The microbial community shifted from an initially diverse flora toward late-stage dominance by *Latilactobacillus sakei*, *L. curvatus*, *Hafnia alvei*, *Serratia* spp., *Carnobacterium maltaromaticum* and *Brochothrix thermosphacta*, while *Candida zeylanoides* persisted throughout storage. TVC exceeded 7 log CFU/g, and TVB-N increased from 10.65 to 23.20 mg N/100 g (p<0.05). TVB-N showed strong positive correlations with all microbial groups ($r_{s}\geq 0.90$, p<0.01) and with seven microbial families at the family level. Hafniaceae dominance coincided with a transient mid-storage decrease in pH, consistent with the deaminative activity of *H. alvei*. $b_{in}^{*}$ showed significant associations with four microbial families and with both microbial counts and TVB-N, supporting its value as a practical spoilage indicator. Sensory evaluation identified Day 13 as the rejection point, corresponding to TVC of 6.79 log CFU/g and TVB-N of 20.80 mg N/100 g, with simultaneous deterioration of odor and appearance, in contrast to the sequential pattern typically reported under aerobic conditions. To our knowledge, this is the first study to integrate time-resolved MALDI-TOF MS-based family-level profiling with physicochemical and sensory monitoring in vacuum-packaged chicken breast stored at 4 °C, offering a condition-specific framework for shelf-life assessment.

## 1. Introduction

Chicken meat is among the most widely consumed animal protein sources worldwide due to its affordability, high nutritional value, and broad consumer acceptance [1]. However, its high water activity, near-neutral pH, and nutrient-rich composition make it highly susceptible to microbial growth and rapid quality deterioration during refrigerated storage. Consequently, microbial spoilage of chilled poultry remains a major challenge for shelf-life management, contributing to economic losses and food waste across the poultry supply chain [2]. The spoilage process of chicken meat has been extensively investigated, and previous studies have demonstrated that microbial succession is strongly influenced by storage temperature, packaging atmosphere, processing hygiene, and initial contamination levels. Under refrigerated poultry storage, Dourou et al. [3] reported that microbial succession is strongly influenced by storage temperature and packaging atmosphere, with psychrotrophic Gram-negative bacteria dominating the early stages of spoilage. Similarly, Doulgeraki et al. [4] demonstrated that oxygen availability substantially alters spoilage ecology by favoring facultative anaerobic taxa under reduced-oxygen conditions. MALDI-TOF MS-based studies by Söylemez Milli [5] and Wang et al. [6] further identified taxa such as *Brochothrix thermosphacta*, *Serratia* spp., *Carnobacterium* spp., Enterobacteriaceae members, and lactic acid bacteria as important spoilage-associated microorganisms in chilled poultry systems. These microorganisms may contribute to off-odors, slime formation, discoloration, pH shifts, and volatile metabolite production, ultimately resulting in sensory rejection of the product [7,8]. Nevertheless, microorganisms reaching high abundance are not always the direct drivers of spoilage. Therefore, the concept of specific spoilage organisms, or, more cautiously, spoilage-associated taxa, remains useful for linking microbial succession to measurable quality deterioration under defined storage conditions [9].

Packaging atmosphere is among the most decisive factors shaping the ecology of poultry spoilage. In aerobic storage systems, *Pseudomonas* spp. frequently dominate, whereas reduced-oxygen conditions may favor *Brochothrix thermosphacta*, facultative anaerobic Enterobacteriaceae, *Carnobacterium* spp., and lactic acid bacteria [4,10]. Vacuum packaging (VP) is therefore widely used in poultry processing because it reduces oxygen availability, limits oxidative reactions, and can extend shelf life under refrigerated conditions [11]. However, VP does not eliminate spoilage risk; rather, it alters microbial ecology and selects for different spoilage consortia compared with aerobic packaging. Previous studies have shown that the packaging atmosphere can substantially influence microbial succession and sensory shelf life in chicken meat, and MALDI-TOF MS has been successfully applied to identify viable isolates in packaged poultry systems [12,13]. Although bacterial populations are often emphasized, yeasts may also persist under chilled low-oxygen storage conditions and contribute to aroma changes, surface growth, or microbial interactions depending on storage ecology and product type [14,15].

Microbial spoilage should be interpreted in conjunction with concurrent physicochemical quality changes rather than solely on microbiological counts. Parameters such as pH, water activity ($a_{w}$), color coordinates ($L^{*}$, $a^{*}$, $b^{*}$), and total volatile basic nitrogen (TVB-N) are widely used indicators of freshness and shelf-life status in poultry meat [16,17,18]. However, each parameter reflects only one aspect of deterioration, and no single indicator can universally define shelf-life termination across all packaging systems and storage scenarios. Therefore, integrated approaches combining microbial ecology with conventional quality indicators are increasingly recommended for more realistic shelf-life evaluation [8,12].

In addition to conventional microbiological counts and physicochemical indicators, several complementary analytical approaches have been applied to characterize spoilage in poultry meat. Culture-independent sequencing methods such as next-generation sequencing (NGS) provide broad insight into microbial community composition without the need for cultivation. Dourou et al. [3] applied 16S rRNA amplicon sequencing to chicken breast and thigh fillets stored under different refrigeration temperatures and demonstrated that temperature substantially shapes succession dynamics. However, DNA-based methods cannot reliably distinguish viable from non-viable cells or persistent extracellular DNA, which may lead to overestimation of metabolically inactive taxa [19,20]. Quantitative PCR (qPCR) offers a targeted alternative capable of rapidly quantifying specific spoilage taxa, including *Pseudomonas* spp., Enterobacteriaceae, and *Brochothrix thermosphacta* directly in meat matrices without cultivation [21]. Biochemical marker approaches represent another dimension of spoilage assessment. Biogenic amines such as putrescine and cadaverine accumulate progressively during microbial proteolysis and deamination, and their concentrations have been linked to spoilage-associated taxa in chilled chicken [22]. TVB-N, measured in the present study, reflects the same underlying biochemical processes at a broader level [17]. Volatile organic compound profiling by gas chromatography–mass spectrometry or proton transfer reaction mass spectrometry can track metabolite-driven spoilage progression, as demonstrated for lactic acid bacteria in modified atmosphere-packaged beef [23] and for packaging-dependent volatile dynamics in chicken breast [24]. Sensor-based platforms integrating machine learning have also shown potential for rapid non-destructive quality screening [25]. Among these approaches, MALDI-TOF MS provides species-level identification of the viable metabolically active fraction at per-sample costs considerably lower than sequencing-based methods and with substantially higher throughput than conventional biochemical identification [14,26,27], making it particularly suitable for time-resolved spoilage monitoring under defined packaging conditions. Recent studies have further demonstrated the utility of MALDI-TOF MS for characterizing spoilage microbiota in vacuum-packaged poultry [28,29,30].

Culture-dependent approaches remain particularly relevant for spoilage studies because they directly target the viable, metabolically active microbial fraction responsible for quality deterioration. In contrast, DNA-based community sequencing, although powerful for resolving overall community composition, does not by itself distinguish viable cells from non-viable cells or persistent extracellular DNA and may therefore overestimate the contribution of taxa that are no longer metabolically active in the food matrix [19,20]. Conventional phenotypic identification methods are labor-intensive and often limited at species level [31]. Matrix-Assisted Laser Desorption/Ionization Time-of-Flight Mass Spectrometry (MALDI-TOF MS) has emerged as a rapid and reliable tool for species-level identification of culturable isolates recovered from food matrices [14,26]. Comparative studies in poultry and meat-associated matrices have reported substantial concordance between MALDI-TOF MS and 16S rRNA or full-length sequencing identification for the dominant culturable taxa [14,26,32], supporting MALDI-TOF MS as a stand-alone, accurate identification approach for the live spoilage fraction rather than only as an exploratory rapid screening method. Although microbial spoilage of poultry meat has been widely studied, comparatively fewer studies have combined time-resolved viable isolate identification by MALDI-TOF MS with conventional microbiological counts and physicochemical quality monitoring in vacuum-packaged chicken breast under controlled refrigerated storage. This limits condition-specific interpretation of spoilage progression and practical shelf-life assessment [14,32].

Accordingly, this study investigated the temporal dynamics of viable culturable microbiota and quality changes in vacuum-packaged chicken breast meat stored at 4 °C for 15 days. Microbial composition and quality indicators were evaluated together using conventional microbiological analyses, MALDI-TOF MS-based species identification, physicochemical measurements, and sensory evaluation. The relationships between microbial groups and quality parameters were also examined to better interpret spoilage progression under vacuum refrigeration. It was hypothesized that vacuum-packaged chicken breast under chilled storage would undergo a structured microbial succession traceable by MALDI-TOF MS, and that shifts in the viable microbiota would be associated with measurable physicochemical and sensory deterioration.

To our knowledge, this is the first study to integrate time-resolved MALDI-TOF MS-based family-level profiling with physicochemical and sensory monitoring in vacuum-packaged chicken breast stored at 4 °C, offering a condition-specific framework for shelf-life assessment. The scientific significance of this approach lies in its potential to identify condition-specific spoilage indicators and rejection thresholds applicable to shelf-life management of vacuum-packaged poultry, and to establish whether the number of monitoring parameters could be reduced in future studies without loss of interpretive power.

## 2. Materials and Methods

### 2.1. Sample Collection and Packaging

Fresh chicken breast fillets (*pectoralis major* muscle) were obtained post-slaughter after complete rigor mortis from a commercial poultry processing plant (Erpiliç, Bolu, Türkiye), where samples had been chilled to 4 °C in accordance with standard slaughterhouse procedures. All samples were taken from a single production lot and transported within 1 h under refrigerated conditions (4 °C) to the Scientific, Industrial and Technological Application and Research Center (SITARC) of Bolu Abant İzzet Baysal University, where VP was performed under aseptic conditions. VP was performed using a vacuum sealer (Lipovak MV-20, Sakarya, Türkiye). Approximately 100 g of chicken breast portions (approximately 5×5×3 cm) were placed in polyamide/polyethene (PA/PE) bags with an oxygen transmission rate (OTR) of 0.33 cm^3^/m^2^/24 h at 23 °C and 50% relative humidity. For each sampling day (0, 1, 2, 3, 4, 5, 7, 9, 13, and 15), two independently vacuum-packaged samples were prepared and analyzed as biological replicates. All samples were stored at 4 ± 0.5 °C in a temperature-controlled refrigerator, and temperature was continuously monitored using a calibrated data logger (Testo 175-T2, Titisee-Neustadt, Germany). This temperature represents the maximum permitted storage temperature for fresh poultry under EU cold chain regulations and is the most widely applied reference condition in poultry spoilage research [2,3,4,10].

### 2.2. Microbiological Analysis

Ten grams of chicken breast were aseptically weighed and homogenized with 90 mL of Maximum Recovery Diluent (MRD; Merck Cat. No. 1.12535, Germany) using a stomacher (Smasher, BioMérieux, Marcy-l’Étoile, France) at 200 rpm for 100 s. Serial decimal dilutions up to 10^−8^ were prepared using the same diluent. Microbiological analyses were performed using two independent vacuum packages per sampling day (biological replicates), and each appropriate dilution was plated in duplicate (technical replicates). Total viable counts (TVC) were determined on Plate Count Agar (PCA; Merck, Cat. No. 1.05463, Germany) after incubation at 30 °C for 48 h. Lactic acid bacteria (LAB) were enumerated on de Man, Rogosa and Sharpe Agar (MRS; Merck, Cat. No. 1.10660, Darmstadt, Germany) at 30 °C for 72 h. Yeasts and molds were counted on Potato Dextrose Agar (PDA; Merck, Cat. No. 1.10130, Germany) at 28 °C for 5 days, and Enterobacteriaceae on Violet Red Bile Agar (VRBA; Merck, Cat. No. 1.10275, Germany) at 37 °C for 24 h. Plates containing 20–200 colonies were used for enumeration, and results were expressed as log_10_ CFU/g [14,33].

### 2.3. Identification of Microorganisms by MALDI-TOF MS

Culturable isolates recovered from the analyzed media were identified by MALDI-TOF MS. For each sampling day, up to 70 colonies per agar type (PCA, MRS, VRBA, and PDA) across appropriate dilution steps were randomly selected for analysis. When different colony morphologies were observed, representative colonies from each visible morphotype were included whenever possible to improve coverage of culturable diversity. Analyses were performed using an Autoflex Speed MALDI-TOF MS instrument (Bruker Daltonics, Bremen, Germany) following the manufacturer’s expanded direct transfer method. Each colony was smeared onto an MTP 384 Ground Steel Target plate and air-dried, after which 1 µL of 70% formic acid and 1 µL of α-cyano-4-hydroxycinnamic acid (α-CHCA; 10 mg/mL) matrix were applied sequentially and allowed to dry. Mass spectra were acquired in linear positive-ion mode with laser intensity set to 50–60% and processed using BioTyper 3.4 software (Bruker Daltonics, Germany). Instrument calibration was performed using the Bruker Bacterial Test Standard (BTS), which contains eight reference peaks (2000–20,000 Da). Identification scores ≥2.00 were accepted as reliable species-level identifications, whereas scores of 1.70–1.99 were considered reliable genus-level identifications [26,34].

### 2.4. Physicochemical Analyses

#### 2.4.1. pH

Ten grams of chicken breast were homogenized with 90 mL of distilled water using a homogenizer (Ultra-Turrax T25, IKA, Staufen im Breisgau, Germany) for 60 s. The pH of each homogenate was measured in duplicate using a calibrated digital pH meter (Orion Star A211, Thermo Scientific, Waltham, MA, USA) [10].

#### 2.4.2. Water Activity ($a_{w}$)

$a_{w}$ was determined at 25 ± 1 °C using a LabMaster $a_{w}$ instrument (Novasina, Lachen, Switzerland) calibrated with standard salt solutions ($a_{w}=0.75$ and 0.90). Measurements were performed in duplicate for each independently packaged sample [2].

#### 2.4.3. Color Measurement

CIE $L^{*}$ (lightness), $a^{*}$ (redness/greenness) and $b^{*}$ (yellowness/blueness) values were measured on both the outer and inner surfaces of each sample using a portable colorimeter (Chroma Meter CR-210, Minolta, Osaka, Japan) calibrated with a white standard plate. The outer surface refers to the external surface of the intact fillet in direct contact with the packaging material. The inner surface refers to the two fresh cut surfaces obtained by bisecting the fillet longitudinally with a sterile knife. Three readings per surface were taken, and mean values were used for analysis [12,18].

#### 2.4.4. Total Volatile Basic Nitrogen (TVB-N)

TVB-N was determined according to the method of Goulas and Kontominas [35], with minor modifications. Ten grams of chicken breast were homogenized with 100 mL of distilled water (Conair^TM^ Waring^TM^ Laboratory Blender, McConnellsburg, PA, USA) and transferred to Kjeldahl digestion tubes with 2 g magnesium oxide (MgO) and a few drops of antifoaming reagent. Steam distillation was performed using a Kjeldahl apparatus (VELP UDK 139, Usmate Velate, Italy); 200 mL of distillate was collected into 25 mL of 3% boric acid containing Tashiro’s indicator, and the distillate was titrated with 0.1 N HCl. Results were expressed as mg N/100 g (duplicate determinations).

#### 2.4.5. Sensory Analysis

Sensory evaluation was conducted on Days 0, 3, 7, 9, 13, and 15 by a trained panel of eight assessors with prior experience in sensory evaluation of meat products. Prior to the study, panelists received training sessions covering the identification and scoring of spoilage-related attributes in raw vacuum-packaged chicken meat, including off-odor development, discoloration, and slime formation, using reference samples at defined spoilage stages. Evaluations were conducted in individual booths under white fluorescent lighting in a dedicated sensory analysis laboratory in accordance with ISO 8589 conditions. For each sampling day, uniform cubes were randomly cut from both vacuum-packaged samples, plated on white dishes coded with randomized three-digit numbers, and presented simultaneously to all eight panelists immediately after removal from packaging. Panelists evaluated samples independently without communication, and each session lasted approximately 15 min. A five-point descriptive intensity scale was used (1 = no detectable spoilage; 5 = complete spoilage). Four attributes were assessed: odor, appearance/color, surface texture/slime, and overall acceptability. The rejection threshold was defined as a mean panel score ≥3.0 for overall acceptability, or ≥3 for odor or appearance individually [7,12]. Attribute means were calculated across all eight panelists per sampling day. Sensory attribute definitions, descriptors, and the rating scale used are provided in Supplementary Table S2, adapted from Katiyo et al. [8].

### 2.5. Statistical Analyses

All physicochemical and microbiological data were analyzed using SPSS Statistics v28.0 (IBM Corp., Armonk, NY, USA). Two independently vacuum-packaged samples per sampling day served as biological replicates. Microbiological enumerations were performed in duplicate platings, and pH, $a_{w}$, and TVB-N were measured in duplicate, yielding n=4 per parameter per day. Surface color was measured at three points per surface, yielding n=6 per surface per day. Microbiological counts were log_10_-transformed prior to analysis. The effect of storage time was assessed by one-way ANOVA with sampling day as the fixed factor. Significant effects (p<0.05) were followed by Tukey’s HSD post hoc test. Results are reported as mean ± SD. For MALDI-TOF MS, up to 70 colonies per medium per sampling day were selected to represent visible morphotypes. Family-level relative abundances were calculated as the proportion of isolates assigned to each family per day. Spearman’s rank correlation coefficients ($r_{s}$) were calculated across the 10 sampling days to assess associations among physicochemical and microbiological parameters and between family-level relative abundances and quality indicators. As sampling days represent temporally dependent observations and multiple parameter pairs were tested without correction for multiple comparisons, reported correlations should be interpreted as exploratory descriptors of co-occurrence patterns rather than confirmatory tests of causal relationships.

## 3. Results and Discussion

### 3.1. Microbial Growth

**Figure 1:**
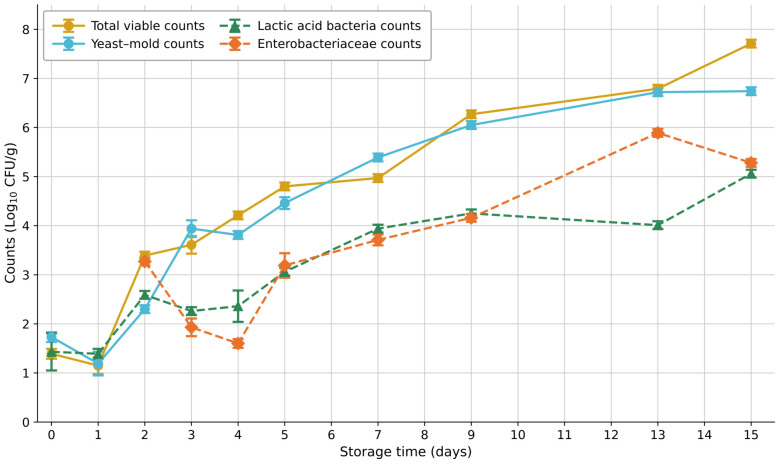
Changes in bacterial counts on different microbiological growth media of VP chicken breast meat samples stored at 4 °C.

All monitored microbial groups increased significantly during 15 days of storage at 4 °C (p<0.05) (Figure 1). Specifically, TVC rose from 1.39 to 7.71 log CFU/g, exceeding the commonly accepted spoilage threshold of 7 log CFU/g on day 15. Yeast–mold counts followed a similar trajectory, reaching 6.74 log CFU/g by day 15. Additionally, LAB increased steadily from 1.43 to 5.06 log CFU/g over the course of storage. In contrast, Enterobacteriaceae showed a non-monotonic pattern, rising to 3.27 log CFU/g on day 2, declining transiently to 1.60 log CFU/g on day 4, then increasing progressively to 5.28 log CFU/g by day 15. A slight decline from 5.89 log CFU/g on Day 13 to 5.28 log CFU/g on Day 15 was also noted, which may reflect competitive suppression by the expanding LAB population during late storage [4,36]. The transient decline likely reflects competitive suppression by the expanding oxygen-tolerant flora combined with the inhibitory effect of oxygen restriction on facultative anaerobic Enterobacteriaceae during early storage [4,15].

The overall growth dynamics are consistent with vacuum-packaged poultry studies reporting suppression of strictly aerobic spoilers and selective enrichment of facultative anaerobic and oxygen-tolerant populations, including LAB [15,37,38]. Samples remained within the microbiologically acceptable range until day 13 based on the 7 log CFU/g threshold [5,12,39]. When the more conservative 6 log CFU/g threshold is applied, however, quality deterioration would be detected earlier [2,6]. This underscores the importance of integrating microbial counts with physicochemical and sensory indicators rather than relying solely on TVC.

A notable finding was the substantial proliferation of yeasts, with yeast–mold counts approaching TVC values by the end of storage. Yeasts have been detected in refrigerated poultry under VP, with gradual increases reported throughout storage [39,40]. In poultry meat systems, yeasts contribute to quality deterioration through alcohol and ester production, off-odor formation, and surface sliminess, while also interacting with bacterial communities [41]. Their persistence here is consistent with tolerance to low temperature and reduced oxygen availability and is further supported by the repeated recovery of *Candida zeylanoides* (*C. zeylanoides*) across multiple sampling days (Section 3.2).

### 3.2. Species-Level Dynamics of Culturable Microbiota Revealed by MALDI-TOF MS

**Figure 2:**
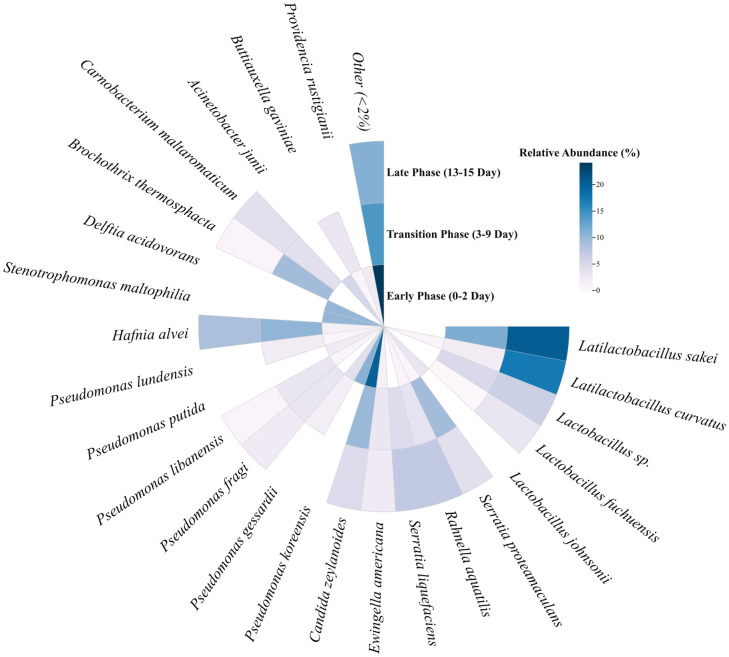
Circular heatmap showing phase-dependent changes in the culturable microbiota of VP chicken breast during refrigerated storage (4 °C). Microbial families identified by MALDI-TOF MS were grouped into Early (0–2 days), Transition (3–9 days), and Late (13–15 days) phases.

**Figure 3:**
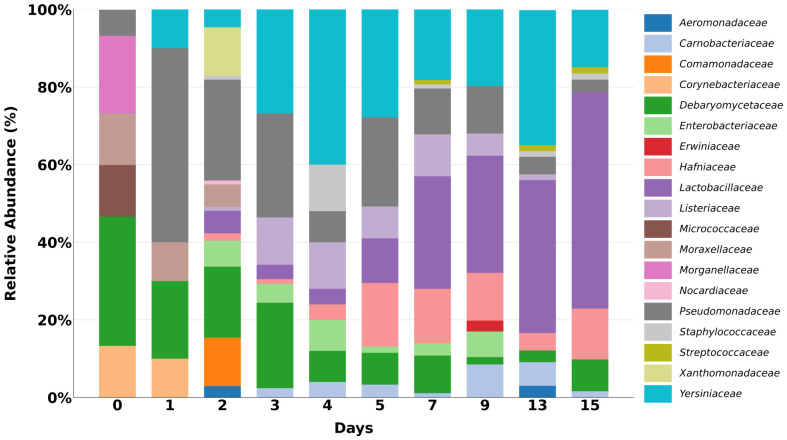
Culture-dependent relative abundance of microbial families in VP chicken breast during storage at 4 °C, determined by MALDI-TOF MS.

**Figure 4:**
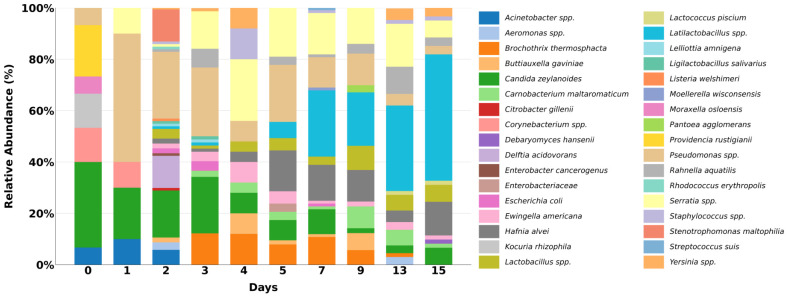
Culture-dependent relative abundance of microbial genera in VP chicken breast during storage at 4 °C, determined by MALDI-TOF MS.

Pure colonies recovered from four culture media across the storage period were identified at species level by MALDI-TOF MS (Supplementary Table S1). A total of 625 isolates corresponded to 33 genera and 67 species-level identifications within 19 families, revealing a rich but temporally variable culturable microbial profile (Figure 2, Figure 3 and Figure 4). The most frequently recovered taxa included *C. zeylanoides* (68 isolates), *Latilactobacillus sakei* (68 isolates; formerly *Lactobacillus sakei*, reclassified by [42]), *Hafnia alvei* (51 isolates), *Serratia proteamaculans* (40 isolates), and *Brochothrix thermosphacta* (35 isolates). These profiles reflect temporal changes in the composition of culturable isolates and should not be interpreted as absolute microbial abundances.

Isolate recovery patterns indicated a progressive shift from psychrotrophic Gram-negative taxa (bacteria that thrive at low temperatures) during early storage toward LAB-associated Gram-positive groups (lactic acid bacteria, which are important spoilage organisms) and selected facultative anaerobic taxa (bacteria that can survive with or without oxygen) during later storage. Debaryomycetaceae, represented predominantly by *C. zeylanoides*, a yeast species, was repeatedly detected at all sampling points. Zhu et al. [15] reported that microbial community composition in meat products undergoes progressive simplification during storage. Similar succession patterns have been described by Dourou et al. [3] and Doulgeraki et al. [4] for chilled poultry stored under different packaging conditions. Comparable MALDI-TOF MS-based microbial profiles were also reported by Augustyńska-Prejsnar et al. [12] for refrigerated chicken breast.

During early storage (Days 0–2), the flora was heterogeneous, with psychrotrophic Gram-negative species typical of environmental or processing sources, including *Pseudomonas* spp., *Acinetobacter* spp., Enterobacteriaceae members, and *Kocuria rhizophila* (Micrococcaceae) (Figure 2, Figure 3 and Figure 4). Sporadic single-isolate identifications should be interpreted cautiously, as MALDI-TOF MS discrimination may be limited among closely related taxa depending on database coverage. This profile is consistent with previous reports on the microbiota of fresh poultry [3,26,43].

As storage progressed (Days 3–9), facultative anaerobic Gram-negative taxa and LAB-associated Gram-positive flora became more prominent, consistent with oxygen-restricted conditions (Figure 2, Figure 3 and Figure 4). *Serratia* spp. (*S. proteamaculans*, *S. liquefaciens* and *S. fonticola*) and *Hafnia alvei* were more frequently recovered, while *Latilactobacillus curvatus*, *L. sakei*, *Ligilactobacillus salivarius*, *Lactobacillus johnsonii* and unidentified *Lactobacillus* spp. were repeatedly detected on consecutive days. In the present study, *Carnobacterium maltaromaticum* and *Brochothrix thermosphacta* were first recovered on Day 3 and persisted until Day 15, consistent with their well-documented roles in the spoilage flora of vacuum- and CO_2_-packaged meat systems, as previously reported by Pothakos et al. [36], Hansen et al. [44], and Lorenzo and Gómez [38].

During late storage (Days 13–15), the profile became less diverse. It concentrated around fewer recurrent taxa, including *Hafnia alvei*, *Serratia* spp., *Latilactobacillus sakei*, *L. curvatus*, *Lactococcus piscium*, *Carnobacterium maltaromaticum* and *Brochothrix thermosphacta* (Figure 2, Figure 3 and Figure 4). *C. zeylanoides* persisted throughout, suggesting strong adaptation to chilled low-oxygen meat environments. Although *Candida zeylanoides* is not considered a primary spoilage organism, it contributes to quality deterioration through the production of short-chain alcohols, esters, and volatile compounds associated with off-odors, and through biofilm-forming interactions with co-existing bacterial communities that may enhance overall spoilage activity [7,40,41]. The increase in TVB-N and microbial counts from Day 13 onward (Figure 1, Table 1) coincided with recurrent recovery of Lactobacillaceae, Hafniaceae, *Serratia* spp. and *Brochothrix thermosphacta*, supporting their candidacy as spoilage-associated taxa under VP conditions [32,45].

Overall, MALDI-TOF MS profiling revealed an initially heterogeneous flora that became progressively simplified through ecological selection under vacuum refrigeration, with LAB-associated taxa, *Hafnia alvei*, *Serratia* spp., and *B. thermosphacta* dominating later stages, alongside persistent *C. zeylanoides*. This succession pattern is consistent with MALDI-TOF MS and sequencing-based reports in meat and poultry systems [3,7,15,32]. Selected studies applying MALDI-TOF MS for spoilage microbiota identification in poultry and related meat systems are summarized in Table 4.

### 3.3. pH

pH is a widely used indicator of freshness in poultry meat. It may be influenced by microbial metabolism and protein degradation during storage [17]. The initial pH of vacuum-packaged chicken breast was 6.15 and showed a non-monotonic pattern throughout storage (p<0.05) (Table 1). Values declined from 6.15 to 5.99 between Days 0 and 5. They dropped sharply to 5.80 on Day 7, then recovered to 6.02–6.11 between Days 9 and 13. The pH decreased again to 5.83 on Day 15. This fluctuating profile reflects competing microbial activities rather than a single directional process. The pH drop on Day 7 coincided with peak Hafniaceae recovery (Days 5–9) (Figure 3 and Figure 4). This is consistent with the strong negative Spearman correlation between Hafniaceae relative abundance and pH ($r_{s}=-0.82$, p<0.01) (Table 3). It supports the role of *H. alvei* in amino acid catabolism and ammonia release during mid-storage [6,46]. The partial pH recovery on Days 9–13 may reflect the growing dominance of Lactobacillaceae. Their fermentative metabolism limits further alkalinization, and CO_2_ dissolves into meat moisture under vacuum [10,36]. Despite fluctuations, pH remained below the 6.3 quality reference for chicken meat [47], which agrees with previous studies on vacuum-packaged chicken [32,48]. The decrease to 5.83 on Day 15 occurred with elevated TVC and TVB-N (Table 1). This confirms that pH alone is insufficient to define spoilage status under VP.

### 3.4. Water Activity ($a_{w}$)

Water activity ($a_{w}$) reflects the availability of free water rather than total moisture content, and remained within a narrow range of 0.923–0.913 throughout storage despite statistically significant differences (p<0.05) (Table 1). The slightly lower values on Days 4–5 (0.913) likely reflect matrix-related variation rather than quality deterioration [49]. Similar stability has been reported for vacuum-packaged poultry [2,17]. Because $a_{w}$ showed minimal variation and did not correlate with spoilage or shelf-life changes in this study, its usefulness as a shelf-life indicator was limited, and spoilage assessment therefore relied on TVB-N, pH, and color.

### 3.5. Color Measurement

Statistically significant changes were observed in $L^{*}$, $a^{*}$, and $b^{*}$ values on both outer and inner surfaces during storage (p<0.05) (Table 1). Measurements on both surfaces provided insight into spatial color variation during storage. Both $L_{out}^{*}$ and $L_{in}^{*}$ declined progressively. This reflects a gradual loss of brightness linked to pigment transformation, lipid oxidation, and increased microbial activity [12,18,50]. The $L_{out}^{*}$ peak on Day 7 (63.14) deviates from the general decreasing trend. It likely reflects anatomical heterogeneity in muscle fiber composition, myoglobin distribution, and local adipose tissue content across measurement points [51]. This should be interpreted with caution. Normal $L^{*}$ values in poultry meat are generally reported in the range of 50–56. Values above 56, when accompanied by low pH, may indicate PSE-like conditions [52,53]. As pH stayed above 5.8 throughout storage, the observed $L^{*}$ pattern reflects gradual loss of brightness rather than excessive paleness.

A limited but progressive increase in $a_{out}^{*}$ and $a_{in}^{*}$ was observed (Table 1). This aligns with a shift from oxymyoglobin toward deoxy- and metmyoglobin forms under low-oxygen conditions [48,50,53]. The positive correlation between $a_{out}^{*}$ and $a_{in}^{*}$ ($r_{s}=0.75$, p<0.05) (Table 2) confirms that this color shift was a sample-wide phenomenon.

The most pronounced change was the progressive increase in $b^{*}$ values, especially on Days 13 and 15 (Table 1). This indicates advancing yellowing in late storage stages, which is linked to lipid oxidation and storage-related deterioration [2,8,18,54]. $b_{in}^{*}$ showed the strongest correlations with microbial and chemical spoilage indicators (Table 3). This is discussed further in Section 3.8.1. Overall, color changes became most evident during late storage, when microbial counts and TVB-N values also increased (Figure 1, Table 1). These changes reflect progressive deterioration in visual quality, consistent with advancing spoilage.

### 3.6. Total Volatile Basic Nitrogen (TVB-N)

TVB-N increased significantly from 10.65 to 23.20 mg N/100 g during storage (p<0.05) (Table 1), reflecting accumulation of ammonia, amines, and other volatile nitrogen compounds through microbial proteolytic and deaminative activity [17,39]. Reported acceptable thresholds vary in the literature: some studies set the rejection limit at 20 mg N/100 g [55], whereas others set it at 25 mg N/100 g [10,37]. TVB-N exceeded 20 mg N/100 g from Day 13 onwards (20.80 mg N/100 g) and reached 23.20 mg N/100 g by Day 15, indicating advancing chemical spoilage under VP conditions. The 25 mg N/100 g level was not reached even at the end of storage, reinforcing that TVB-N alone does not define shelf-life termination and should be interpreted alongside other quality indicators [8,9,17,39,46]. The strong correlation between TVB-N and microbial counts ($r_{s}\geq 0.90$, p<0.01) (Table 2) is addressed in Section 3.8.1.

### 3.7. Sensory Evaluation

**Figure 5:**
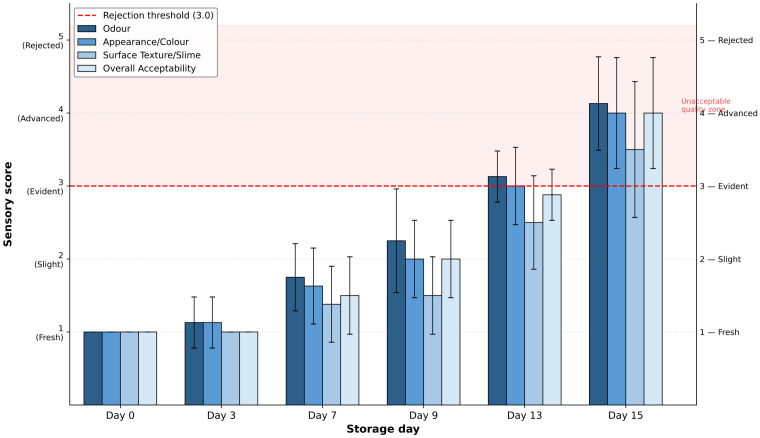
Sensory score profiles of VP chicken breast stored at 4 °C for 15 days, evaluated by a trained panel (n=8) using a five-point descriptive scale. The dashed red line indicates the sensory rejection threshold (score = 3.0). The shaded area above the rejection threshold indicates the unacceptable quality zone.

Sensory scores showed clear, progressive deterioration across all attributes during storage (Figure 5). At Days 0 and 3, mean scores (1.00–1.25) indicated a fully fresh and acceptable product. On Day 7, odor and appearance/color scores rose to 1.75 and 1.63, respectively. However, the product remained acceptable, with TVC (4.97 log CFU/g) and TVB-N (15.80 mg N/100 g) values below rejection thresholds (Figure 1, Table 1).

On Day 9, the mean odor score was 2.25, with two panelists assigning a score of 3, indicating borderline quality. This coincided with TVC of 6.27 log CFU/g and TVB-N of 17.55 mg N/100 g (Figure 1, Table 1), while the overall panel score remained below the rejection threshold. Day 13 represented the rejection point. Mean odor and appearance/color scores reached 3.13 and 3.00, respectively, with all panelists assigning odor scores ≥3, triggering the rejection criterion. This corresponded to a TVC of 6.79 log CFU/g and TVB-N of 20.80 mg N/100 g (Figure 1, Table 1). Concurrent increases in $a_{in}^{*}$ (5.58) and $b_{in}^{*}$ (8.14) (Table 1) reflected instrumental color changes consistent with sensory appearance deterioration. By Day 15, all attributes indicated advanced spoilage. Mean odor score was 4.13, appearance/color 4.00, surface texture/slime 3.50, and overall acceptability 4.00, with panelist P4 assigning the maximum odor score of 5 (Figure 5). These scores coincided with TVC exceeding the 7 log CFU/g spoilage threshold (7.71 log CFU/g) and TVB-N reaching 23.20 mg N/100 g (Figure 1, Table 1).

Surface texture/slime yielded the lowest scores throughout storage, reaching 3.50 only on Day 15. This is consistent with the suppression of aerobic spoilers under VP and agrees with previous reports of slime formation becoming apparent only at TVC exceeding 8 log CFU/g under aerobic conditions [8]. In contrast to aerobic storage, where *Pseudomonas* spp. drive rapid off-odor development that typically precedes appearance changes [8,15], both odor and appearance/color reached the rejection threshold simultaneously on Day 13 in the present study. This parallel rather than sequential deterioration reflects the distinct microbial ecology of VP, where *Pseudomonas* spp. are suppressed, and the concurrent dominance of Lactobacillaceae, Hafniaceae and Carnobacteriaceae generates both volatile metabolites and visible surface changes in a coordinated manner [4,10,15]. Differences in product type (breast fillet versus leg) may also contribute to this pattern.

### 3.8. Correlation Analysis

Spearman correlation coefficients were calculated to assess relationships among physicochemical and microbiological parameters and between family-level relative abundances derived from MALDI-TOF MS and quality indicators (Table 2 and Table 3).

#### 3.8.1. Relationships Between Physicochemical and Microbiological Parameters

TVB-N, an indicator of microbial proteolysis and deamination [7,17], showed strong positive correlations with all microbial groups ($r_{s}=0.90$–0.99, p<0.01) (Table 2). This confirms its role as a primary indicator of volatile nitrogen accumulation in chilled poultry [8,9,32]. TVB-N also correlated positively with $b_{in}^{*}$ ($r_{s}=0.69$, p<0.05) (Table 2). This is consistent with lipid oxidation and heme degradation contributing to inner-surface yellowing [18,50,53,54]. However, lipid oxidation under VP conditions has been reported to remain considerably lower than under aerobic or PVC overwrap packaging [56], suggesting that heme pigment transformation and microbial metabolite production may be the primary drivers of $b_{in}^{*}$ increase in the present study. Microbial pigments, especially from *Serratia* spp., may enhance this effect [32,45]. Associations with $a_{in}^{*}$ ($r_{s}=0.53$, p>0.05) and $L_{in}^{*}$ ($r_{s}=0.33$, p>0.05) were not statistically significant (Table 2).

pH showed weak, non-significant negative relationships with TVB-N ($r_{s}=-0.54$, p>0.05) and microbial counts ($r_{s}=-0.36$ to −0.54, all p>0.05) (Table 2). These values reflect competing processes—acid production by LAB, alkaline metabolite release by Hafniaceae and *Serratia* spp., and CO_2_ dissolution under vacuum conditions [3,6,10,36]. As a result, pH followed a buffered, non-directional trend, even as microbial load increased. This trend is consistent with the buffering capacity of meat proteins [57]. No significant correlations were detected between $a_{w}$ and any other parameter (Table 2), consistent with its narrow and stable range (0.913–0.923) under VP [2,11,17].

A strong negative correlation between $L_{out}^{*}$ and $a_{out}^{*}$ ($r_{s}=-0.76$, p<0.05) (Table 2) indicates simultaneous surface darkening and $a_{out}^{*}$ increase under low-oxygen conditions, consistent with oxymyoglobin conversion to deoxy- and metmyoglobin forms [48,50,53]. The positive $a_{out}^{*}$–$a_{in}^{*}$ correlation ($r_{s}=0.75$, p<0.05) (Table 2) confirms that this color shift occurred throughout the sample.

Among color parameters, $b_{in}^{*}$ was the most informative spoilage descriptor, showing significant correlations with TVB-N ($r_{s}=0.69$, p<0.05), TVC ($r_{s}=0.72$, p<0.05), yeast–mold ($r_{s}=0.69$, p<0.05), LAB ($r_{s}=0.72$, p<0.05) and Enterobacteriaceae ($r_{s}=0.85$, p<0.01) (Table 2). This indicates that inner-surface color changes reflect the combined effects of lipid oxidation, heme degradation, microbial metabolism, and pigment production, supporting $b_{in}^{*}$ as a practical color-based shelf-life indicator for vacuum-packaged poultry [2,8,18,48,53,54].

Sensory evaluation supported these findings. The rejection threshold was reached on Day 13, coinciding with TVC of 6.79 log CFU/g and TVB-N of 20.80 mg N/100 g (Figure 1, Table 1), alongside concurrent increases in $b_{in}^{*}$ (8.14) and $a_{in}^{*}$ (5.58) (Table 1). The simultaneous deterioration of odor and appearance contrasts with the sequential pattern reported under aerobic conditions [8] and reflects coordinated microbial activity under VP.

#### 3.8.2. Relationships Between Microbial Families and Physicochemical Parameters

Family-level correlation analysis based on MALDI-TOF MS identifications provided a mechanistic view of spoilage progression in vacuum-packaged chicken breast (Table 3, Figure 3 and Figure 4). The strongest positive correlation with TVB-N was observed for Lactobacillaceae ($r_{s}=0.96$, p<0.01), followed by Hafniaceae ($r_{s}=0.78$, p<0.01), Streptococcaceae ($r_{s}=0.75$, p<0.05) and Carnobacteriaceae ($r_{s}=0.67$, p<0.05). These taxa are facultative anaerobes or microaerophiles adapted to low-oxygen environments, explaining their selective enrichment under VP [4,36,46].

The Lactobacillaceae–TVB-N association reflects temporal co-occurrence rather than direct contribution, as these bacteria are primarily fermentative and weakly proteolytic [36]. Nevertheless, indirect pathways may contribute to this relationship. LAB fermentation generates organic acids that lower local pH and CO_2_ that dissolves under vacuum conditions, both of which may modulate the metabolic activity of co-occurring proteolytic taxa such as *Hafnia alvei* and *Serratia* spp. [4,17,36]. The main biochemical drivers of TVB-N formation are more plausibly *Hafnia alvei* (Hafniaceae) and *Serratia* spp. (Yersiniaceae), which drive amino acid deamination, biogenic amine production, and trimethylamine N-oxide reduction [3,6,32,45].

Hafniaceae showed the only statistically significant negative correlation with pH ($r_{s}=-0.82$, p<0.01) (Table 3), consistent with its role in mid-storage amino acid catabolism and ammonia release [6,46]. Its peak recovery between Days 5 and 9 (Figure 3 and Figure 4) coincided with the transient pH drop on Day 7 (Table 1), followed by partial recovery as Lactobacillaceae became dominant. Carnobacteriaceae also showed significant positive correlations with TVB-N ($r_{s}=0.67$, p<0.05). Streptococcaceae additionally correlated with $b_{in}^{*}$ ($r_{s}=0.65$, p<0.05), whereas the Carnobacteriaceae–$b_{in}^{*}$ association was not significant ($r_{s}=0.34$, p>0.05) (Table 3), reflecting their contribution to volatile production under vacuum conditions [7,36,38,44].

*Brochothrix thermosphacta* (Listeriaceae) was recovered from Day 3 onwards and persisted until Day 15. Despite the absence of a significant family-level TVB-N correlation ($r_{s}=0.14$, p>0.05) (Table 3), it generates acetoin, isobutyric acid, and short-chain fatty acids characteristic of dairy-like off-odors in chilled meat [7,46] and should be considered a relevant secondary spoilage organism in vacuum-packaged poultry.

Debaryomycetaceae ($r_{s}=-0.78$, p<0.01), Moraxellaceae ($r_{s}=-0.81$, p<0.01), and Corynebacteriaceae ($r_{s}=-0.70$, p<0.05) showed negative correlations with TVB-N (Table 3), indicating association with early storage before advanced spoilage. The negative Debaryomycetaceae correlation reflects the declining relative proportion of *C. zeylanoides* as bacterial taxa dominated the culturable community during late storage, rather than its absence. *C. zeylanoides* nonetheless contributes to spoilage through short-chain alcohols, esters, and biofilm-forming interactions with bacteria [7,40,41]. Moraxellaceae, comprising obligate aerobic psychrotrophs, progressively lost competitiveness under oxygen restriction [3,26]. The positive correlation between Moraxellaceae and $a_{w}$ ($r_{s}=0.63$, p<0.05) (Table 3) further supports their association with early-storage, surface-aerobic conditions.

Pseudomonadaceae showed strong negative correlations with $b_{out}^{*}$ ($r_{s}=-0.84$, p<0.01) and $b_{in}^{*}$ ($r_{s}=-0.69$, p<0.05) (Table 3), consistent with their progressive displacement under oxygen restriction and replacement by facultative anaerobic taxa driving late-stage yellowing [3,11,26]. Conversely, Lactobacillaceae ($r_{s}=0.80$, p<0.01), Streptococcaceae ($r_{s}=0.65$, p<0.05) and Staphylococcaceae ($r_{s}=0.66$, p<0.05) correlated positively with $b_{in}^{*}$ (Table 3), confirming that inner-surface yellowing is linked to the late-stage facultative anaerobic consortium.

Overall, the family-level correlation matrix indicates a coherent ecological transition from early aerobic communities (Moraxellaceae, Corynebacteriaceae, Pseudomonadaceae) to VP-adapted facultative anaerobes (Lactobacillaceae, Hafniaceae, Carnobacteriaceae, Streptococcaceae) and persistent yeasts (Debaryomycetaceae), accompanied by progressive TVB-N accumulation, transient mid-storage acidification and inner-surface yellowing, providing a mechanistic interpretation of spoilage progression in vacuum-packaged chicken breast at 4 °C. Despite its analytical utility, the translation of the MALDI-TOF MS-based framework to routine industrial or regulatory screening faces practical constraints (Table 4). Instrument investment costs remain substantial, and per-sample workflows are culture-dependent, requiring 24–48 h incubation before identification is possible [27]. DNA-based alternatives face their own limitations, as they cannot reliably distinguish viable from non-viable cells [19]. Identification accuracy depends on database coverage, and taxa with limited representation may yield unreliable scores [26]. At the regulatory level, MALDI-TOF MS-based spoilage profiling has not yet been integrated into standardized frameworks. Future studies should validate this approach across multiple production batches and facilities, examine additional packaging formats and storage temperatures, and explore integration with rapid sensor-based screening and predictive modeling tools [14,26,27].

## 4. Conclusions

Storage of vacuum-packaged chicken breast at 4 °C for 15 days resulted in a progressive spoilage trajectory driven by coupled microbial and physicochemical changes. MALDI-TOF MS identified 625 culturable isolates belonging to 67 species across 19 families, revealing a shift from an initially diverse community toward a late-stage flora dominated by *Latilactobacillus sakei*, *L. curvatus*, *Hafnia alvei*, *Serratia* spp., *Carnobacterium maltaromaticum* and *Brochothrix thermosphacta*, while *C. zeylanoides* persisted throughout storage. TVB-N increased from 10.65 to 23.20 mg N/100 g and showed strong positive correlations with all microbial groups ($r_{s}\geq 0.90$, p<0.01), with Lactobacillaceae, Hafniaceae, Carnobacteriaceae and Streptococcaceae identified as the primary spoilage-associated families under vacuum conditions. Among physicochemical parameters, inner-surface yellowness ($b_{in}^{*}$) emerged as the most practical spoilage indicator, while pH and $a_{w}$ showed limited discriminatory value. Hafniaceae was the only family negatively correlated with pH, consistent with the deaminative activity of *H. alvei*. Sensory rejection occurred on Day 13 at TVC of 6.79 log CFU/g and TVB-N of 20.80 mg N/100 g, with simultaneous deterioration of odor and appearance, in contrast to the sequential pattern typically observed under aerobic conditions. These findings demonstrate that integrating MALDI-TOF MS with microbiological, physicochemical, and sensory indicators provides a condition-specific framework for shelf-life assessment in vacuum-packaged poultry. Future studies incorporating multiple production batches and complementary volatile organic compound profiling would further strengthen mechanistic interpretation.

## Figures and Tables

**Table 1:** Physicochemical and color values (mean ± SD) in vacuum-packaged chicken breast meat during refrigerated storage.

Day	TVB-N	pH	*a_w_*	Outer Surface			Inner Surface		
				$L_{\mathrm{out}}^{*}$	$a_{\mathrm{out}}^{*}$	$b_{\mathrm{out}}^{*}$	$L_{\mathrm{in}}^{*}$	$a_{\mathrm{in}}^{*}$	$b_{\mathrm{in}}^{*}$
0	10.65 ± 0.21 ^*j*^	6.15 ± 0.01 ^*a*^	0.923 ± 0.001 ^*a*^	60.51 ± 2.06 ^*a*^	2.33 ± 0.06 ^*c*^	6.78 ± 1.66 ^*a*^	56.78 ± 1.96 ^*b*^	2.69 ± 0.07 ^*c*^	4.90 ± 0.68 ^*c*^
1	11.25 ± 0.21 ^*i*^	6.07 ± 0.02 ^*c*^	0.921 ± 0.001 ^*a*^	57.91 ± 0.42 ^*b*^	2.60 ± 0.33 ^*b*^	4.30 ± 1.45 ^*b*^	56.96 ± 1.76 ^*b*^	3.01 ± 0.31 ^*c*^	3.72 ± 0.83 ^*d*^
2	11.95 ± 0.13 ^*h*^	6.04 ± 0.02 ^*c*^	0.923 ± 0.001 ^*a*^	57.40 ± 1.21 ^*b*^	2.96 ± 0.62 ^*b*^	5.02 ± 0.71 ^*a*^	57.52 ± 0.26 ^*b*^	2.50 ± 0.54 ^*c*^	6.00 ± 0.07 ^*b*^
3	12.40 ± 0.16 ^*g*^	6.05 ± 0.02 ^*c*^	0.919 ± 0.001 ^*b*^	58.20 ± 1.85 ^*b*^	3.54 ± 0.08 ^*a*^	4.92 ± 0.27 ^*b*^	58.57 ± 2.32 ^*b*^	3.40 ± 0.15 ^*b*^	4.44 ± 0.75 ^*c*^
4	13.60 ± 0.18 ^*f*^	6.01 ± 0.01 ^*d*^	0.913 ± 0.001 ^*c*^	57.50 ± 0.26 ^*b*^	3.87 ± 0.43 ^*a*^	5.53 ± 0.19 ^*a*^	57.38 ± 0.83 ^*b*^	2.71 ± 0.60 ^*c*^	4.94 ± 0.27 ^*c*^
5	14.40 ± 0.18 ^*e*^	5.99 ± 0.01 ^*d*^	0.913 ± 0.001 ^*c*^	59.50 ± 2.29 ^*b*^	2.13 ± 0.23 ^*c*^	5.45 ± 0.77 ^*a*^	57.74 ± 0.81 ^*b*^	1.64 ± 0.07 ^*d*^	4.56 ± 0.43 ^*c*^
7	15.80 ± 0.16 ^*d*^	5.80 ± 0.01 ^*e*^	0.920 ± 0.001 ^*a*^	63.14 ± 0.30 ^*a*^	2.01 ± 0.04 ^*c*^	5.34 ± 0.41 ^*a*^	58.67 ± 0.42 ^*b*^	2.67 ± 0.51 ^*c*^	4.94 ± 0.25 ^*c*^
9	17.55 ± 0.21 ^*c*^	6.02 ± 0.02 ^*d*^	0.923 ± 0.002 ^*a*^	56.10 ± 1.04 ^*c*^	3.61 ± 0.16 ^*a*^	4.57 ± 1.35 ^*b*^	57.81 ± 1.25 ^*b*^	3.96 ± 0.19 ^*b*^	5.93 ± 0.43 ^*b*^
13	20.80 ± 0.16 ^*b*^	6.11 ± 0.01 ^*b*^	0.916 ± 0.001 ^*b*^	59.45 ± 0.96 ^*b*^	3.82 ± 0.14 ^*a*^	5.50 ± 0.76 ^*a*^	63.84 ± 0.98 ^*a*^	5.58 ± 0.30 ^*a*^	8.14 ± 0.37 ^*a*^
15	23.20 ± 0.16 ^*a*^	5.83 ± 0.02 ^*e*^	0.918 ± 0.002 ^*b*^	55.69 ± 1.75 ^*c*^	3.91 ± 0.54 ^*a*^	5.95 ± 0.76 ^*a*^	55.87 ± 0.40 ^*c*^	5.12 ± 0.40 ^*a*^	6.33 ± 0.40 ^*b*^

Different superscript lowercase letters within the same column indicate significantly different means (p<0.05; Tukey’s HSD). Data are mean ± SD (n=4 for TVB-N, pH, $a_{w}$; n=6 for color values). TVB-N: total volatile basic nitrogen (mg N/100 g); $a_{w}$: water activity.

**Table 2:** Spearman correlation coefficients ($r_{s}$) between physicochemical parameters, color values, and microbiological counts in vacuum-packaged chicken breast meat (n=10 sampling days).

	TVB-N	pH	$a_{w}$	$L_{\mathrm{out}}^{*}$	$a_{\mathrm{out}}^{*}$	$b_{\mathrm{out}}^{*}$	$L_{\mathrm{in}}^{*}$	$a_{\mathrm{in}}^{*}$	$b_{\mathrm{in}}^{*}$	TVC	Y-M	LAB
**TVB-N**												
**pH**	−0.54											
$a_{w}$	−0.49	+0.41										
$L_{\mathrm{out}}^{*}$	−0.28	+0.15	−0.05									
$a_{\mathrm{out}}^{*}$	+0.48	+0.02	−0.27	−0.76 *								
$b_{\mathrm{out}}^{*}$	+0.16	−0.04	−0.25	+0.18	+0.22							
$L_{\mathrm{in}}^{*}$	+0.33	−0.03	−0.21	+0.39	−0.19	−0.36						
$a_{\mathrm{in}}^{*}$	+0.53	+0.25	−0.07	−0.43	+0.75 *	+0.01	+0.09					
$b_{\mathrm{in}}^{*}$	+0.68 *	−0.18	−0.04	−0.35	+0.50	+0.36	+0.18	+0.39				
**TVC**	+0.99 **	−0.52	−0.45	−0.24	+0.47	+0.27	+0.32	+0.50	+0.72 *			
**Y-M**	+0.98 **	−0.48	−0.41	−0.21	+0.43	+0.21	+0.37	+0.53	+0.68 *	+0.99 **		
**LAB**	+0.94 **	−0.54	−0.26	−0.37	+0.37	+0.01	+0.32	+0.43	+0.72 *	+0.93 **	+0.94 **	
**Entero.**	+0.90 **	−0.36	−0.23	−0.27	+0.39	+0.07	+0.45	+0.47	+0.85 **	+0.90 **	+0.91 **	+0.95 **

* p<0.05; ** p<0.01 (Spearman’s rank correlation coefficients, n=10 sampling days). For Enterobacteriaceae (Entero.), samples below the detection limit (<1 log CFU/g) on days 0 and 1 were assigned a value of 1.0 log CFU/g. TVB-N: total volatile basic nitrogen; $a_{w}$: water activity; Y-M: yeast–mold count.

**Table 3:** Spearman correlation coefficients ($r_{s}$) between microbial families and TVB-N, pH, $a_{w}$ and color values in vacuum-packaged chicken breast meat (n=10 storage days).

Family	TVB-N	pH	$a_{w}$	$L_{\mathrm{out}}^{*}$	$a_{\mathrm{out}}^{*}$	$b_{\mathrm{out}}^{*}$	$L_{\mathrm{in}}^{*}$	$a_{\mathrm{in}}^{*}$	$b_{\mathrm{in}}^{*}$
Aeromonadaceae	0.14	0.37	−0.04	−0.05	0.20	0.03	0.39	0.16	0.63
Carnobacteriaceae	0.67 *	−0.17	−0.47	−0.20	0.49	−0.01	0.50	0.46	0.34
Comamonadaceae	−0.29	0.06	0.29	−0.29	−0.06	−0.17	−0.06	−0.41	0.29
Corynebacteriaceae	−0.70 *	0.59	0.51	0.29	−0.36	0.08	−0.53	−0.10	−0.50
Debaryomycetaceae	−0.78 **	0.39	0.41	0.38	−0.51	−0.02	−0.33	−0.38	−0.61
Enterobacteriaceae	−0.08	−0.29	−0.04	−0.28	0.11	−0.28	0.23	−0.33	0.03
Erwiniaceae	0.29	−0.12	0.41	−0.41	0.17	−0.41	0.17	0.29	0.18
Hafniaceae	0.78 **	−0.82 **	−0.45	0.03	−0.04	0.15	0.33	−0.10	0.38
Lactobacillaceae	0.96 **	−0.54	−0.28	−0.31	0.41	0.18	0.30	0.42	0.80 **
Listeriaceae	0.14	−0.29	−0.43	0.23	−0.04	−0.19	0.63 *	−0.15	−0.20
Lysobacteraceae	−0.29	0.06	0.23	−0.29	−0.06	−0.17	−0.06	−0.41	0.29
Micrococcaceae	−0.52	0.53	0.53	0.41	−0.29	0.52	−0.40	−0.17	−0.18
Moraxellaceae	−0.81 **	0.59	0.63 *	0.13	−0.37	−0.02	−0.53	−0.31	−0.32
Morganellaceae	−0.31	0.08	0.51	0.68 *	−0.59	0.39	−0.06	−0.34	−0.14
Nocardiaceae	−0.29	0.06	0.29	−0.29	−0.06	−0.17	−0.06	−0.41	0.29
Pseudomonadaceae	−0.56	0.11	0.18	0.02	−0.44	−0.84 **	0.14	−0.39	−0.69 *
Staphylococcaceae	0.51	−0.35	−0.49	−0.25	0.58	0.51	−0.03	0.25	0.66 *
Streptococcaceae	0.75 *	−0.32	−0.26	−0.02	0.32	0.40	0.14	0.50	0.65 *
Yersiniaceae	0.49	−0.19	−0.80 **	0.03	0.36	0.04	0.52	0.24	0.11

* p<0.05; ** p<0.01 (Spearman correlation coefficients, n=10 storage days). TVB-N: total volatile basic nitrogen; $a_{w}$: water activity.

**Table 4:** Comparative overview of studies applying MALDI-TOF MS for spoilage microbiota identification in poultry meat under different storage and packaging conditions.

Ref.	Product/Packaging	Storage Conditions	MALDI-TOF MS Approach	Main Spoilage Taxa	Relevance to Present Study
[26]	Skinless chicken breast. High O_2_ MAP (80%/20% CO_2_); Low O_2_ MAP (65% N_2_/35% CO_2_)	4 and 10 °C, 14 days	MALDI-TOF MS from culture plates	*B. thermosphacta*, *Carnobacterium* spp., *Pseudomonas* spp. (High O_2_); *Carnobacterium* spp., *Serratia* spp., *H. alvei* (Low O_2_, 4 °C)	Resolved spoilage dynamics at species level in MAP poultry; taxa under reduced-O_2_ overlap with present study
[14]	Chicken carcass (breast skin), aerobic, retail	7 and 30 °C, cross-sectional	MALDI-TOF MS + 16S rRNA sequencing	*Pseudomonas* spp. dominant, *Brochothrix*, *Psychrobacter*	High concordance between MALDI-TOF MS and 16S rRNA; supports culture-dependent MALDI-TOF MS for active spoilage fraction
[28]	Minced free-range chicken; VP and MAP (30% CO_2_/70% N_2_)	4 °C, 10 days	MALDI-TOF MS, VP vs. MAP spoilage microbiota	VP: LAB (*Carnobacterium* spp., Lactobacillaceae), Enterobacteriaceae; MAP: *B. thermosphacta* and LAB	VP at 4 °C confirms dominance of *Carnobacterium* and LAB under reduced-O_2_
[29]	Chicken breast (*M. pectoralis major*), MAP, retail	4 and 8 °C, use-by date +4 d	MALDI-TOF MS, 120 isolates; TVC, Enterobacteriaceae, LAB	*C. divergens*, *C. maltaromaticum*, *B. thermosphacta*, *H. alvei*, *S. proteamaculans*	Taxa shared with present study confirmed at species level
[30]	Minced poultry hamburgers, MAP ± sulfites	Chilled, shelf-life trial	MALDI-TOF MS + TVC, pathogen detection	*Carnobacterium* spp. dominant, *H. alvei* at final spoilage	*Carnobacterium* spp. and *H. alvei* confirmed; CO_2_ shapes microbiota consistent with VP findings
[12]	Broiler chicken breast, aerobic	0.5 °C, 12 days	MALDI-TOF MS + sensory evaluation, 468 isolates	*P. fragi*, *P. lundensis*, *P. gessardii* dominant from day 8	Provided MALDI-TOF MS and sensory data; present study extends to VP with physicochemical monitoring
[24]	Korat chicken breast fillets, aerobic/VP/MAP	4 °C, shelf-life trial	MALDI-TOF MS + VOC profiling	*Pseudomonas* spp. (aerobic), LAB and Enterobacteriaceae (VP/MAP)	Packaging atmosphere shapes spoilage microbiota and VOC profiles consistent with present study
[5]	Chicken breast fillet, aerobic (overwrap)	4 °C, 13 days	MALDI-TOF MS + microbiological + physicochemical	Early: *Acinetobacter* spp., *Candida* spp.; final: LAB, *Serratia* spp., *Hafnia* spp.	Aerobic spoilage at 4 °C contrasts with VP succession; highlights packaging-driven shift
**Present study**	Chicken breast fillet, VP	4 °C, 15 days, 10 sampling days	MALDI-TOF MS + TVC, LAB, Y-M, Entero., pH, $a_{w}$, TVB-N, CIE $L^{*}a^{*}b^{*}$, sensory panel; 625 isolates, 67 species, 19 families	*L. sakei*, *L. curvatus*, *H. alvei*, *Serratia* spp., *C. maltaromaticum*, *B. thermosphacta*, *C. zeylanoides*	First integrated time-resolved MALDI-TOF MS + physicochemical + sensory framework for VP chicken breast at 4 °C

MAP: modified atmosphere packaging; VP: vacuum packaging; LAB: lactic acid bacteria; TVB-N: total volatile basic nitrogen; $a_{w}$: water activity; VOC: volatile organic compounds; TVC: total viable count; Y-M: yeast–mold count.

## Data Availability

The data presented in this study are available on request from the corresponding author. Supplementary Table S1 contains the complete list of MALDI-TOF MS isolate identifications. Supplementary Table S2 provides sensory attribute definitions, descriptors, and reference standards. The sensory analysis data are also available upon request.

## Supplementary Materials

The following supporting information can be downloaded at: https://www.mdpi.com/article/10.3390/foods15122162/s1, Table S1: Complete list of MALDI-TOF MS isolate identifications (625 isolates across 10 sampling days); Table S2: Sensory attribute definitions, descriptors and reference standards adapted for vacuum-packaged chicken breast.

## Abbreviations

The following abbreviations are used in this manuscript:

$a_{w}$	water activity
LAB	lactic acid bacteria
MALDI-TOF MS	Matrix-Assisted Laser Desorption/Ionization Time-of-Flight Mass Spectrometry
MRD	Maximum Recovery Diluent
MRS	de Man, Rogosa and Sharpe agar
OTR	oxygen transmission rate
PA/PE	polyamide/polyethylene
PCA	Plate Count Agar
PDA	Potato Dextrose Agar
TVB-N	total volatile basic nitrogen
TVC	total viable count
VOC	volatile organic compounds
VP	vacuum packaging
VRBA	Violet Red Bile Agar
