# Supplementary material for: Integrated MALDI-TOF MS, Microbiological, Physicochemical and Sensory Assessment of Spoilage in Vacuum-Packaged Chicken Breast During Refrigerated Storage
Source: Foods. 2026 Jun 15;15(12):2162. doi: 10.3390/foods15122162 (PMC13298445; doi:10.3390/foods15122162)
Supplement: Supplementary file 1 [file foods-15-02162-s001.zip › foods-4329653-supplementary.pdf]

Supplementary Table S1: Putative identities of culturable isolates obtained from VP chicken breast meat during refrigerated storage (4 °C), determined by MALDI-TOF MS. Isolate IDs encode the sampling day (0, 1, 2, 3, 4, 5, 7, 9, 13, 15) and the isolation medium: P = Plate Count Agar (PCA), M = de Man, Rogosa and Sharpe agar (MRS), V = Violet Red Bile Agar (VRBA), and PD = Potato Dextrose Agar (PDA). MALDI-TOF MS identification scores are reported for each isolate.

Sampling day	Isolate ID	MALDI-TOF MS ID	MALDI-TOF MS Score
Day-0	M01	*Candida zeylanoides*	1.914
Day-0	M02	*Candida zeylanoides*	2.020
Day-0	M03	*Corynebacterium variabile*	1.894
Day-0	M04	*Candida zeylanoides*	1.954
Day-0	P01	*Corynebacterium xerosis*	2.045
Day-0	P02	*Kocuria rhizophila*	2.073
Day-0	P03	*Providencia rustigianii*	2.227
Day-0	P04	*Kocuria rhizophila*	2.222
Day-0	P05	*Providencia rustigianii*	2.305
Day-0	P06	*Providencia rustigianii*	2.233
Day-0	PD01	*Candida zeylanoides*	1.722
Day-0	PD02	*Moraxella osloensis*	1.922
Day-0	PD03	*Pseudomonas coagulans*	1.966
Day-0	PD04	*Candida zeylanoides*	1.834
Day-0	PD05	*Acinetobacter johnsonii*	2.163
Day-1	P11	*Candida zeylanoides*	1.740
Day-1	P12	*Corynebacterium falsenii*	2.148
Day-1	P13	*Pseudomonas gessardii*	2.331
Day-1	P14	*Acinetobacter junii*	2.045
Day-1	P15	*Candida zeylanoides*	1.746
Day-1	P16	*Pseudomonas gessardii*	2.184
Day-1	P17	*Pseudomonas kilonensis*	2.131
Day-1	P18	*Pseudomonas lundensis*	1.911
Day-1	P19	*Pseudomonas taetrolens*	2.182
Day-1	P110	*Serratia fonticola*	1.738
Day-2	M21	*Lactobacillus johnsonii*	2.099
Day-2	M22	*Candida zeylanoides*	2.166
Day-2	M23	*Candida zeylanoides*	2.023
Day-2	M24	*Candida zeylanoides*	1.760
Day-2	M25	*Lactobacillus johnsonii*	2.218
Day-2	M26	*Candida zeylanoides*	2.081
Day-2	M27	*Ligilactobacillus salivarius*	2.059
Day-2	M28	*Lactobacillus johnsonii*	2.157
Day-2	M29	*Latilactobacillus curvatus*	2.236
Day-2	M210	*Candida zeylanoides*	1.905
Day-2	M211	*Candida zeylanoides*	1.889
Day-2	M212	*Candida zeylanoides*	1.900
Day-2	M213	*Lactobacillus johnsonii*	2.192
Day-2	M214	*Candida zeylanoides*	1.871
Day-2	M215	*Candida zeylanoides*	1.731
Day-2	M216	*Candida zeylanoides*	1.914
Day-2	M217	*Candida zeylanoides*	1.957
Day-2	P21	*Delftia acidovorans*	2.243
Day-2	P22	*Pseudomonas koreensis*	2.248
Day-2	P23	*Pseudomonas koreensis*	2.259
Day-2	P24	*Delftia acidovorans*	2.419
Day-2	P25	*Enterobacter cancerogenus*	1.762
Day-2	P26	*Aeromonas popoffii*	2.190
Day-2	P27	*Delftia acidovorans*	1.977
Day-2	P28	*Delftia acidovorans*	2.232
Day-2	P29	*Ewingella americana*	1.703
Day-2	P210	*Listeria welshimeri*	2.150
Day-2	P211	*Aeromonas bestiarum*	1.771
Day-2	P212	*Stenotrophomonas maltophilia*	2.109
Day-2	P213	*Delftia acidovorans*	2.270

Day-2	P214	*Pseudomonas koreensis*	2.063
Day-2	P215	*Stenotrophomonas maltophilia*	2.240
Day-2	P216	*Pseudomonas koreensis*	2.190
Day-2	P217	*Stenotrophomonas maltophilia*	2.103
Day-2	P218	*Yersinia enterocolitica*	1.892
Day-2	P219	*Pseudomonas fluorescens*	1.942
Day-2	P220	*Acinetobacter junii*	2.038
Day-2	P221	*Delftia acidovorans*	2.288
Day-2	P222	*Stenotrophomonas maltophilia*	1.964
Day-2	P223	*Delftia acidovorans*	2.221
Day-2	P224	*Delftia acidovorans*	1.937
Day-2	P225	*Pseudomonas koreensis*	1.993
Day-2	P226	*Pseudomonas gessardii*	2.087
Day-2	P227	*Delftia acidovorans*	2.271
Day-2	P228	*Staphylococcus equorum*	2.050
Day-2	P229	*Pseudomonas azotoformans*	1.780
Day-2	P230	*Pseudomonas koreensis*	1.975
Day-2	P231	*Stenotrophomonas maltophilia*	1.824
Day-2	P232	*Acinetobacter junii*	2.220
Day-2	P233	*Buttiauxella gaviniae*	1.727
Day-2	P234	*Pseudomonas libanensis*	1.979
Day-2	P235	*Citrobacter gillenii*	2.194
Day-2	P236	*Buttiauxella gaviniae*	2.059
Day-2	P237	*Stenotrophomonas maltophilia*	1.996
Day-2	V21	*Escherichia coli*	1.776
Day-2	V22	*Acinetobacter junii*	1.721
Day-2	V23	*Delftia acidovorans*	1.771
Day-2	V24	*Delftia acidovorans*	2.317
Day-2	V25	*Delftia acidovorans*	1.887
Day-2	V26	*Pseudomonas koreensis*	2.048
Day-2	V27	*Stenotrophomonas maltophilia*	1.964
Day-2	V28	*Ewingella americana*	1.861
Day-2	V29	*Escherichia coli*	2.164
Day-2	V210	*Pseudomonas koreensis*	2.210
Day-2	V211	*Pseudomonas koreensis*	1.873
Day-2	V212	*Hafnia alvei*	1.970
Day-2	V213	*Pseudomonas koreensis*	1.800
Day-2	V214	*Pseudomonas corrugata*	1.955
Day-2	V215	*Stenotrophomonas maltophilia*	2.067
Day-2	V216	*Stenotrophomonas maltophilia*	1.899
Day-2	V217	*Stenotrophomonas maltophilia*	1.869
Day-2	V218	*Acinetobacter junii*	2.040
Day-2	V219	*Pseudomonas koreensis*	2.032
Day-2	V220	*Hafnia alvei*	2.331
Day-2	V221	*Rahnella aquatilis*	1.849
Day-2	V222	*Delftia acidovorans*	1.834
Day-2	V223	*Pseudomonas koreensis*	2.250
Day-2	PD21	*Pseudomonas aeruginosa*	1.793
Day-2	PD22	*Pseudomonas putida*	1.778
Day-2	PD23	*Serratia proteamaculans*	1.721
Day-2	PD24	*Pseudomonas putida*	1.744
Day-2	PD25	*Candida zeylanoides*	1.760
Day-2	PD26	*Candida zeylanoides*	1.875
Day-2	PD27	*Pseudomonas putida*	1.984
Day-2	PD28	*Stenotrophomonas maltophilia*	1.933
Day-2	PD29	*Pseudomonas rhodesiae*	1.916
Day-2	PD210	*Candida zeylanoides*	1.769
Day-2	PD211	*Candida zeylanoides*	1.741
Day-2	PD212	*Candida zeylanoides*	1.763
Day-2	PD213	*Candida zeylanoides*	1.725
Day-2	PD214	*Pseudomonas gessardii*	2.026
Day-2	PD215	*Pseudomonas gessardii*	2.045
Day-2	PD216	*Rhodococcus erythropolis*	2.139
Day-2	PD217	*Stenotrophomonas maltophilia*	1.836
Day-2	PD218	*Lelliottia amnigena*	2.174

Day-2	PD219	*Acinetobacter junii*	1.799
Day-2	PD220	*Candida zeylanoides*	1.765
Day-2	PD221	*Candida zeylanoides*	1.882
Day-2	PD222	*Pseudomonas poae*	2.003
Day-2	PD223	*Pseudomonas koreensis*	1.701
Day-2	PD224	*Aeromonas salmonicida*	1.950
Day-2	PD225	*Pseudomonas koreensis*	1.746
Day-2	PD226	*Acinetobacter junii*	2.027
Day-2	PD227	*Stenotrophomonas maltophilia*	2.073
Day-3	P31	*Candida zeylanoides*	1.729
Day-3	P32	*Brochothrix thermosphacta*	1.925
Day-3	P33	*Pseudomonas fragi*	1.991
Day-3	P34	*Brochothrix thermosphacta*	1.735
Day-3	P35	*Pseudomonas fragi*	2.029
Day-3	P36	*Pseudomonas fragi*	2.132
Day-3	P37	*Serratia proteamaculans*	1.774
Day-3	P38	*Serratia proteamaculans*	2.314
Day-3	P39	*Lelliottia amnigena*	1.931
Day-3	P310	*Brochothrix thermosphacta*	1.834
Day-3	P311	*Candida zeylanoides*	1.735
Day-3	P312	*Brochothrix thermosphacta*	1.989
Day-3	P313	*Candida zeylanoides*	1.926
Day-3	P314	*Pseudomonas gessardii*	2.223
Day-3	P315	*Pseudomonas fluorescens*	1.940
Day-3	P316	*Carnobacterium maltaromaticum*	2.294
Day-3	P317	*Pseudomonas libanensis*	2.059
Day-3	P318	*Candida zeylanoides*	1.897
Day-3	P319	*Candida zeylanoides*	1.893
Day-3	P320	*Brochothrix thermosphacta*	2.313
Day-3	P321	*Brochothrix thermosphacta*	2.072
Day-3	P322	*Brochothrix thermosphacta*	2.247
Day-3	P323	*Brochothrix thermosphacta*	2.240
Day-3	P324	*Carnobacterium maltaromaticum*	2.507
Day-3	P325	*Candida zeylanoides*	2.006
Day-3	P326	*Pseudomonas rhodesiae*	1.987
Day-3	P327	*Serratia liquefaciens*	1.731
Day-3	P328	*Rahnella aquatilis*	2.017
Day-3	P329	*Serratia liquefaciens*	1.788
Day-3	P330	*Serratia proteamaculans*	2.203
Day-3	P331	*Pseudomonas gessardii*	2.113
Day-3	P332	*Lactobacillus* sp.	1.748
Day-3	P333	*Pseudomonas libanensis*	2.116
Day-3	P334	*Pseudomonas libanensis*	2.152
Day-3	P335	*Brochothrix thermosphacta*	2.064
Day-3	V31	*Escherichia coli*	1.930
Day-3	V32	*Escherichia coli*	2.253
Day-3	V33	*Rahnella aquatilis*	2.037
Day-3	V34	*Yersinia enterocolitica*	2.265
Day-3	V35	*Rahnella aquatilis*	1.990
Day-3	V36	*Hafnia alvei*	2.417
Day-3	V37	*Rahnella aquatilis*	1.900
Day-3	V38	*Rahnella aquatilis*	1.932
Day-3	V39	*Rahnella aquatilis*	1.999
Day-3	M31	*Candida zeylanoides*	2.090
Day-3	M32	*Candida zeylanoides*	1.814
Day-3	M33	*Latilactobacillus curvatus*	2.040
Day-3	M34	*Ligilactobacillus salivarius*	1.791
Day-3	M35	*Candida zeylanoides*	1.728
Day-3	M36	*Candida zeylanoides*	1.706
Day-3	M37	*Candida zeylanoides*	1.795
Day-3	M38	*Candida zeylanoides*	1.719
Day-3	M39	*Candida zeylanoides*	1.984
Day-3	M310	*Candida zeylanoides*	1.988
Day-3	PD31	*Ewingella americana*	1.718
Day-3	PD32	*Candida zeylanoides*	2.041

Day-3	PD33	*Pseudomonas asplenii*	1.793
Day-3	PD34	*Candida zeylanoides*	1.817
Day-3	PD35	*Serratia proteamaculans*	1.713
Day-3	PD36	*Pseudomonas libanensis*	2.089
Day-3	PD37	*Ewingella americana*	1.737
Day-3	PD38	*Serratia liquefaciens*	2.030
Day-3	PD39	*Escherichia coli*	1.979
Day-3	PD310	*Pseudomonas libanensis*	2.245
Day-3	PD311	*Serratia proteamaculans*	1.795
Day-3	PD312	*Pseudomonas tolaasii*	2.037
Day-3	PD313	*Pseudomonas rhodesiae*	1.857
Day-3	PD314	*Candida zeylanoides*	1.921
Day-3	PD315	*Candida zeylanoides*	1.860
Day-3	PD316	*Pseudomonas libanensis*	1.940
Day-3	PD317	*Pseudomonas lundensis*	2.142
Day-3	PD318	*Serratia liquefaciens*	1.911
Day-3	PD319	*Pseudomonas tolaasii*	2.141
Day-3	PD320	*Serratia proteamaculans*	2.174
Day-3	PD321	*Serratia proteamaculans*	2.340
Day-3	PD322	*Ewingella americana*	1.787
Day-3	PD323	*Pseudomonas libanensis*	2.152
Day-3	PD324	*Pseudomonas libanensis*	2.143
Day-3	PD325	*Pseudomonas gessardii*	2.138
Day-3	PD326	*Serratia proteamaculans*	2.256
Day-3	PD327	*Brochothrix thermosphacta*	1.936
Day-3	PD328	*Pseudomonas mucidolens*	1.898
Day-4	P41	*Carnobacterium maltaromaticum*	2.430
Day-4	P42	*Serratia plymuthica*	1.773
Day-4	P43	*Ewingella americana*	2.310
Day-4	P44	*Staphylococcus epidermidis*	2.141
Day-4	P45	*Staphylococcus epidermidis*	1.938
Day-4	P46	*Brochothrix thermosphacta*	1.973
Day-4	P47	*Pseudomonas libanensis*	1.903
Day-4	P48	*Lactobacillus* sp.	2.375
Day-4	P49	*Candida zeylanoides*	1.887
Day-4	P410	*Brochothrix thermosphacta*	2.113
Day-4	V41	*Hafnia alvei*	2.144
Day-4	V42	*Buttiauxella gaviniae*	2.222
Day-4	V43	*Buttiauxella gaviniae*	2.298
Day-4	V44	*Serratia liquefaciens*	2.199
Day-4	V45	*Yersinia enterocolitica*	1.728
Day-4	V46	*Yersinia enterocolitica*	2.212
Day-4	V47	*Serratia liquefaciens*	2.245
Day-4	M41	*Candida zeylanoides*	1.986
Day-4	PD41	*Brochothrix thermosphacta*	1.766
Day-4	PD42	*Pseudomonas libanensis*	2.176
Day-4	PD43	*Serratia proteamaculans*	1.961
Day-4	PD44	*Serratia plymuthica*	1.717
Day-4	PD45	*Staphylococcus hominis*	2.189
Day-4	PD46	*Ewingella americana*	1.808
Day-4	PD47	*Serratia plymuthica*	1.704
Day-5	P51	*Lactobacillus* sp.	2.385
Day-5	P52	*Serratia proteamaculans*	1.801
Day-5	P53	*Serratia proteamaculans*	1.957
Day-5	P54	*Serratia proteamaculans*	2.206
Day-5	P55	*Ewingella americana*	2.306
Day-5	P56	*Pseudomonas fragi*	1.815
Day-5	P57	*Brochothrix thermosphacta*	1.878
Day-5	P58	*Brochothrix thermosphacta*	1.849
Day-5	P59	*Brochothrix thermosphacta*	2.289
Day-5	P510	*Lactobacillus* sp.	1.733
Day-5	P511	*Carnobacterium maltaromaticum*	2.317
Day-5	P512	*Pseudomonas fragi*	1.899
Day-5	P513	*Serratia proteamaculans*	2.121
Day-5	P514	*Serratia liquefaciens*	2.263

Day-5	P515	*Carnobacterium maltaromaticum*	2.378
Day-5	P516	*Pseudomonas rhodesiae*	2.124
Day-5	P517	*Pseudomonas tolaasii*	1.911
Day-5	P518	*Brochothrix thermosphacta*	1.856
Day-5	P519	*Pseudomonas brenneri*	2.157
Day-5	P520	*Lactobacillus* sp.	1.811
Day-5	P521	*Pseudomonas lundensis*	2.034
Day-5	V51	*Hafnia alvei*	2.424
Day-5	V52	*Serratia liquefaciens*	2.430
Day-5	V53	*Hafnia alvei*	2.301
Day-5	V54	*Hafnia alvei*	2.458
Day-5	V55	*Hafnia alvei*	1.938
Day-5	V56	*Hafnia alvei*	2.190
Day-5	V57	*Hafnia alvei*	2.328
Day-5	V58	*Serratia proteamaculans*	2.289
Day-5	V59	*Hafnia alvei*	2.392
Day-5	V510	*Rahnella aquatilis*	1.805
Day-5	V511	*Rahnella aquatilis*	2.109
Day-5	V512	*Hafnia alvei*	2.430
Day-5	V513	*Serratia proteamaculans*	1.915
Day-5	M51	*Candida zeylanoides*	1.897
Day-5	M52	*Enterobacteriaceae*	2.268
Day-5	M53	*Latilactobacillus curvatus*	2.272
Day-5	M54	*Enterobacteriaceae*	2.065
Day-5	M55	*Latilactobacillus sakei*	2.590
Day-5	M56	*Latilactobacillus sakei*	2.597
Day-5	M57	*Latilactobacillus curvatus*	2.448
Day-5	M58	*Candida zeylanoides*	2.036
Day-5	M59	*Candida zeylanoides*	2.061
Day-5	M510	*Candida zeylanoides*	2.237
Day-5	M511	*Candida zeylanoides*	1.891
Day-5	PD51	*Pseudomonas libanensis*	1.812
Day-5	PD52	*Serratia proteamaculans*	1.886
Day-5	PD53	*Brochothrix thermosphacta*	2.086
Day-5	PD54	*Hafnia alvei*	1.778
Day-5	PD55	*Buttiauxella gaviniae*	1.836
Day-5	PD56	*Serratia proteamaculans*	1.920
Day-5	PD57	*Ewingella americana*	1.738
Day-5	PD58	*Pseudomonas libanensis*	1.880
Day-5	PD59	*Pseudomonas fragi*	1.900
Day-5	PD510	*Pseudomonas fragi*	1.921
Day-5	PD511	*Hafnia alvei*	2.023
Day-5	PD512	*Ewingella americana*	1.755
Day-5	PD513	*Serratia proteamaculans*	1.996
Day-5	PD514	*Serratia proteamaculans*	2.115
Day-5	PD515	*Pseudomonas kilonensis*	1.769
Day-5	PD516	*Pseudomonas taetrolens*	1.911
Day-5	PD517	*Pseudomonas mucidolens*	1.791
Day-5	PD518	*Pseudomonas rhodesiae*	1.744
Day-7	V71	*Latilactobacillus curvatus*	2.247
Day-7	V72	*Latilactobacillus curvatus*	2.165
Day-7	V73	*Latilactobacillus curvatus*	2.202
Day-7	V74	*Candida zeylanoides*	1.927
Day-7	V75	*Hafnia alvei*	2.288
Day-7	V76	*Hafnia alvei*	2.268
Day-7	V77	*Hafnia alvei*	2.405
Day-7	V78	*Buttiauxella gaviniae*	2.222
Day-7	V79	*Hafnia alvei*	2.452
Day-7	V710	*Hafnia alvei*	2.402
Day-7	V711	*Hafnia alvei*	2.352
Day-7	V712	*Hafnia alvei*	2.298
Day-7	V713	*Hafnia alvei*	2.251
Day-7	V714	*Hafnia alvei*	2.150
Day-7	V715	*Hafnia alvei*	2.134
Day-7	V716	*Hafnia alvei*	2.405

Day-7	V717	*Moellerella wisconsensis*	2.251
Day-7	V718	*Escherichia coli*	1.750
Day-7	V719	*Hafnia alvei*	2.446
Day-7	V720	*Serratia liquefaciens*	2.468
Day-7	P71	*Lactobacillus sp.*	1.760
Day-7	P72	*Brochothrix thermosphacta*	1.749
Day-7	P73	*Serratia plymuthica*	1.732
Day-7	P74	*Serratia proteamaculans*	2.117
Day-7	P75	*Brochothrix thermosphacta*	1.824
Day-7	P76	*Brochothrix thermosphacta*	1.855
Day-7	P77	*Serratia proteamaculans*	2.098
Day-7	P78	*Brochothrix thermosphacta*	2.050
Day-7	P79	*Pseudomonas lundensis*	1.956
Day-7	P710	*Pseudomonas lundensis*	2.074
Day-7	P711	*Pseudomonas lundensis*	2.122
Day-7	P712	*Brochothrix thermosphacta*	1.869
Day-7	P713	*Brochothrix thermosphacta*	2.068
Day-7	P714	*Serratia proteamaculans*	2.001
Day-7	P715	*Brochothrix thermosphacta*	1.972
Day-7	P716	*Brochothrix thermosphacta*	2.154
Day-7	P717	*Pseudomonas fragi*	2.262
Day-7	P718	*Brochothrix thermosphacta*	2.003
Day-7	P719	*Pseudomonas fragi*	1.797
Day-7	P720	*Streptococcus suis*	1.956
Day-7	P721	*Carnobacterium maltaromaticum*	2.485
Day-7	P722	*Serratia proteamaculans*	2.028
Day-7	P723	*Brochothrix thermosphacta*	1.777
Day-7	P724	*Candida zeylanoides*	2.158
Day-7	P725	*Lactobacillus sp.*	1.830
Day-7	P726	*Pseudomonas lundensis*	2.236
Day-7	P727	*Serratia liquefaciens*	1.845
Day-7	P728	*Serratia proteamaculans*	2.334
Day-7	P729	*Lactobacillus* sp.	2.450
Day-7	P730	*Candida zeylanoides*	2.042
Day-7	M71	*Latilactobacillus sakei*	2.153
Day-7	M72	*Latilactobacillus sakei*	2.390
Day-7	M73	*Latilactobacillus sakei*	2.467
Day-7	M74	*Latilactobacillus sakei*	2.532
Day-7	M75	*Latilactobacillus sakei*	2.550
Day-7	M76	*Latilactobacillus sakei*	2.367
Day-7	M77	*Candida zeylanoides*	1.841
Day-7	M78	*Latilactobacillus sakei*	2.394
Day-7	M79	*Latilactobacillus sakei*	2.425
Day-7	M710	*Latilactobacillus sakei*	1.836
Day-7	M711	*Latilactobacillus sakei*	2.122
Day-7	M712	*Latilactobacillus sakei*	2.347
Day-7	M713	*Candida zeylanoides*	1.743
Day-7	M714	*Latilactobacillus sakei*	2.303
Day-7	M715	*Latilactobacillus sakei*	2.282
Day-7	M716	*Latilactobacillus sakei*	2.189
Day-7	M717	*Latilactobacillus sakei*	2.284
Day-7	M718	*Latilactobacillus sakei*	2.349
Day-7	M719	*Lactobacillus fuchuensis*	2.183
Day-7	M720	*Latilactobacillus sakei*	2.436
Day-7	M721	*Latilactobacillus curvatus*	1.921
Day-7	M722	*Candida zeylanoides*	2.047
Day-7	M723	*Latilactobacillus sakei*	2.283
Day-7	M724	*Latilactobacillus sakei*	2.200
Day-7	M725	*Staphylococcus hominis*	2.263
Day-7	M726	*Candida zeylanoides*	1.810
Day-7	PD71	*Pseudomonas fragi*	1.982
Day-7	PD72	*Pseudomonas lundensis*	1.799
Day-7	PD73	*Pseudomonas lundensis*	1.951
Day-7	PD74	*Serratia liquefaciens*	1.827
Day-7	PD75	*Serratia liquefaciens*	2.043

Day-7	PD76	*Pseudomonas taetrolens*	1.918
Day-7	PD77	*Serratia proteamaculans*	2.266
Day-7	PD78	*Serratia liquefaciens*	1.905
Day-7	PD79	*Rahnella aquatilis*	1.741
Day-7	PD710	*Ewingella americana*	1.957
Day-7	PD711	*Serratia proteamaculans*	1.873
Day-7	PD712	*Pseudomonas azotoformans*	1.796
Day-7	PD713	*Candida zeylanoides*	2.007
Day-7	PD714	*Serratia proteamaculans*	2.078
Day-7	PD715	*Hafnia alvei*	1.960
Day-7	PD716	*Serratia liquefaciens*	1.821
Day-7	PD717	*Candida zeylanoides*	1.767
Day-9	V91	*Serratia fonticola*	1.980
Day-9	V92	*Serratia fonticola*	2.264
Day-9	V93	*Hafnia alvei*	2.225
Day-9	V94	*Buttiauxella gaviniae*	2.145
Day-9	V95	*Serratia liquefaciens*	2.281
Day-9	V96	*Serratia liquefaciens*	2.364
Day-9	V97	*Buttiauxella gaviniae*	1.974
Day-9	V98	*Hafnia alvei*	2.394
Day-9	V99	*Hafnia alvei*	2.307
Day-9	V910	*Rahnella aquatilis*	2.075
Day-9	V911	*Hafnia alvei*	2.494
Day-9	V912	*Hafnia alvei*	2.427
Day-9	V913	*Hafnia alvei*	2.251
Day-9	V914	*Serratia fonticola*	2.256
Day-9	V915	*Hafnia alvei*	2.392
Day-9	V916	*Hafnia alvei*	2.263
Day-9	V917	*Rahnella aquatilis*	1.970
Day-9	V918	*Hafnia alvei*	2.357
Day-9	V919	*Buttiauxella gaviniae*	2.302
Day-9	V920	*Hafnia alvei*	2.425
Day-9	V921	*Rahnella aquatilis*	2.055
Day-9	V922	*Hafnia alvei*	2.181
Day-9	V923	*Hafnia alvei*	2.492
Day-9	V924	*Hafnia alvei*	2.081
Day-9	P91	*Lactobacillus sp.*	2.398
Day-9	P92	*Buttiauxella gaviniae*	2.113
Day-9	P93	*Carnobacterium maltaromaticum*	2.414
Day-9	P94	*Lactobacillus* sp.	2.390
Day-9	P95	*Brochothrix thermosphacta*	2.166
Day-9	P96	*Lactobacillus* sp.	1.822
Day-9	P97	*Brochothrix thermosphacta*	2.252
Day-9	P98	*Pseudomonas fragi*	1.825
Day-9	P99	*Serratia plymuthica*	1.721
Day-9	P910	*Serratia proteamaculans*	2.282
Day-9	P911	*Brochothrix thermosphacta*	2.098
Day-9	P912	*Lactobacillus* sp.	2.422
Day-9	P913	*Pseudomonas fragi*	2.357
Day-9	P914	*Buttiauxella gaviniae*	2.283
Day-9	P915	*Serratia proteamaculans*	2.293
Day-9	P916	*Brochothrix thermosphacta*	2.192
Day-9	P917	*Serratia proteamaculans*	2.285
Day-9	P918	*Serratia liquefaciens*	1.781
Day-9	P919	*Lactobacillus* sp.	1.809
Day-9	P920	*Brochothrix thermosphacta*	2.199
Day-9	P921	*Lactobacillus* sp.	2.436
Day-9	P922	*Carnobacterium maltaromaticum*	2.275
Day-9	P923	*Lactobacillus* sp.	2.444
Day-9	P924	*Pseudomonas gessardii*	2.028
Day-9	P925	*Ewingella americana*	2.060
Day-9	P926	*Lactobacillus* sp.	1.800
Day-9	P927	*Carnobacterium maltaromaticum*	2.400
Day-9	P928	*Pseudomonas taetrolens*	2.107
Day-9	P929	*Serratia proteamaculans*	2.290

Day-9	P930	*Serratia plymuthica*	1.883
Day-9	P931	*Ewingella americana*	2.212
Day-9	P932	*Carnobacterium maltaromaticum*	2.298
Day-9	P933	*Serratia proteamaculans*	2.276
Day-9	P934	*Carnobacterium maltaromaticum*	2.409
Day-9	P935	*Carnobacterium maltaromaticum*	2.380
Day-9	P936	*Lactobacillus* sp.	2.447
Day-9	P937	*Pseudomonas rhodesiae*	2.053
Day-9	P938	*Carnobacterium maltaromaticum*	2.385
Day-9	P939	*Carnobacterium maltaromaticum*	2.396
Day-9	P940	*Brochothrix thermosphacta*	2.269
Day-9	P941	*Lactobacillus* sp.	2.127
Day-9	P942	*Carnobacterium maltaromaticum*	2.483
Day-9	P943	*Rahnella aquatilis*	2.109
Day-9	M91	*Latilactobacillus sakei*	2.496
Day-9	M92	*Latilactobacillus sakei*	2.490
Day-9	M93	*Latilactobacillus sakei*	2.436
Day-9	M94	*Latilactobacillus sakei*	2.323
Day-9	M95	*Latilactobacillus sakei*	2.095
Day-9	M96	*Latilactobacillus sakei*	2.520
Day-9	M97	*Latilactobacillus sakei*	2.391
Day-9	M98	*Latilactobacillus sakei*	2.484
Day-9	M99	*Candida zeylanoides*	2.048
Day-9	M910	*Latilactobacillus sakei*	2.426
Day-9	M911	*Latilactobacillus sakei*	2.288
Day-9	M912	*Latilactobacillus sakei*	2.115
Day-9	M913	*Latilactobacillus sakei*	2.047
Day-9	M914	*Latilactobacillus sakei*	2.352
Day-9	M915	*Latilactobacillus sakei*	2.545
Day-9	M916	*Latilactobacillus sakei*	2.539
Day-9	M917	*Latilactobacillus sakei*	2.562
Day-9	M918	*Latilactobacillus sakei*	2.557
Day-9	M919	*Latilactobacillus curvatus*	2.355
Day-9	M920	*Latilactobacillus sakei*	2.467
Day-9	M921	*Latilactobacillus sakei*	2.511
Day-9	M922	*Latilactobacillus sakei*	2.544
Day-9	M923	*Candida zeylanoides*	2.091
Day-9	M924	*Latilactobacillus sakei*	2.173
Day-9	PD91	*Pantoea agglomerans*	2.040
Day-9	PD92	*Pantoea agglomerans*	1.705
Day-9	PD93	*Pantoea agglomerans*	2.081
Day-9	PD94	*Serratia proteamaculans*	1.958
Day-9	PD95	*Pseudomonas mucidolens*	1.717
Day-9	PD96	*Pseudomonas mucidolens*	1.771
Day-9	PD97	*Pseudomonas poae*	1.722
Day-9	PD98	*Pseudomonas gessardii*	1.829
Day-9	PD99	*Pseudomonas gessardii*	1.744
Day-9	PD910	*Pseudomonas gessardii*	2.039
Day-9	PD911	*Pseudomonas taetrolens*	1.859
Day-9	PD912	*Serratia proteamaculans*	1.722
Day-9	PD913	*Buttiauxella gaviniae*	1.724
Day-9	PD914	*Pseudomonas mucidolens*	1.778
Day-9	PD915	*Buttiauxella gaviniae*	1.840
Day-13	V131	*Serratia liquefaciens*	2.171
Day-13	V132	*Serratia liquefaciens*	2.379
Day-13	V133	*Yersinia pseudotuberculosis*	1.851
Day-13	V134	*Serratia liquefaciens*	2.297
Day-13	V135	*Serratia liquefaciens*	1.972
Day-13	V136	*Yersinia enterocolitica*	2.229
Day-13	V137	*Serratia liquefaciens*	1.932
Day-13	V138	*Serratia liquefaciens*	2.240
Day-13	V139	*Rahnella aquatilis*	1.945
Day-13	V1310	*Hafnia alvei*	2.339
Day-13	V1311	*Rahnella aquatilis*	1.718
Day-13	V1312	*Aeromonas bestiarum*	2.001

Day-13	V1313	*Hafnia alvei*	2.150
Day-13	V1314	*Yersinia ruckeri*	2.091
Day-13	V1315	*Rahnella aquatilis*	1.849
Day-13	V1316	*Rahnella aquatilis*	1.743
Day-13	V1317	*Rahnella aquatilis*	1.714
Day-13	V1318	*Hafnia alvei*	2.185
Day-13	P131	*Lactobacillus* sp.	2.380
Day-13	P132	*Carnobacterium maltaromaticum*	2.377
Day-13	P133	*Rahnella aquatilis*	1.928
Day-13	P134	*Serratia plymuthica*	1.775
Day-13	P135	*Serratia liquefaciens*	1.706
Day-13	P136	*Lactobacillus* sp.	2.438
Day-13	P137	*Lactobacillus* sp.	2.350
Day-13	P138	*Aeromonas bestiarum*	2.254
Day-13	P139	*Ewingella americana*	1.855
Day-13	P1310	*Rahnella aquatilis*	1.798
Day-13	P1311	*Lactobacillus* sp.	2.409
Day-13	P1312	*Serratia proteamaculans*	1.905
Day-13	P1313	*Staphylococcus hominis*	2.268
Day-13	M131	*Latilactobacillus sakei*	2.544
Day-13	M132	*Latilactobacillus sakei*	2.480
Day-13	M133	*Latilactobacillus curvatus*	2.286
Day-13	M134	*Latilactobacillus curvatus*	2.360
Day-13	M135	*Latilactobacillus curvatus*	2.291
Day-13	M136	*Candida zeylanoides*	2.050
Day-13	M137	*Latilactobacillus sakei*	2.428
Day-13	M138	*Latilactobacillus sakei*	2.561
Day-13	M139	*Latilactobacillus curvatus*	2.038
Day-13	M1310	*Latilactobacillus curvatus*	2.116
Day-13	M1311	*Latilactobacillus sakei*	2.596
Day-13	M1312	*Latilactobacillus sakei*	2.355
Day-13	M1313	*Latilactobacillus sakei*	2.583
Day-13	M1314	*Latilactobacillus sakei*	2.443
Day-13	M1315	*Latilactobacillus curvatus*	2.005
Day-13	M1316	*Candida zeylanoides*	1.983
Day-13	M1317	*Latilactobacillus sakei*	2.508
Day-13	M1318	*Latilactobacillus sakei*	2.630
Day-13	M1319	*Latilactobacillus sakei*	2.626
Day-13	M1320	*Latilactobacillus sakei*	2.467
Day-13	M1321	*Latilactobacillus sakei*	2.310
Day-13	M1322	*Latilactobacillus sakei*	2.438
Day-13	M1323	*Latilactobacillus sakei*	2.442
Day-13	M1324	*Latilactobacillus sakei*	2.527
Day-13	PD131	*Pseudomonas libanensis*	1.963
Day-13	PD132	*Carnobacterium maltaromaticum*	1.725
Day-13	PD133	*Pseudomonas taetrolens*	1.806
Day-13	PD134	*Serratia proteamaculans*	1.945
Day-13	PD135	*Ewingella americana*	1.969
Day-13	PD136	*Carnobacterium maltaromaticum*	2.259
Day-13	PD137	*Carnobacterium maltaromaticum*	2.270
Day-13	PD138	*Serratia proteamaculans*	2.135
Day-13	PD139	*Brochothrix thermosphacta*	1.906
Day-13	PD1310	*Pseudomonas fragi*	2.010
Day-13	PD1311	*Lactococcus piscium*	2.032
Day-15	V151	*Yersinia ruckeri*	1.918
Day-15	V152	*Hafnia alvei*	2.256
Day-15	V153	*Hafnia alvei*	2.341
Day-15	V154	*Hafnia alvei*	2.196
Day-15	V155	*Hafnia alvei*	2.390
Day-15	V156	*Rahnella aquatilis*	2.026
Day-15	V157	*Rahnella aquatilis*	1.801
Day-15	V158	*Hafnia alvei*	2.394
Day-15	V159	*Serratia liquefaciens*	2.455
Day-15	V1510	*Serratia liquefaciens*	2.338
Day-15	V1511	*Hafnia alvei*	2.065

Day-15	V1512	*Yersinia enterocolitica*	1.893
Day-15	V1513	*Ewingella americana*	2.123
Day-15	V1514	*Hafnia alvei*	2.261
Day-15	P151	*Lactobacillus* sp.	1.726
Day-15	P152	*Hafnia alvei*	2.429
Day-15	P153	*Carnobacterium maltaromaticum*	2.467
Day-15	P154	*Lactobacillus sp.*	1.763
Day-15	PD151	*Lactococcus piscium*	1.858
Day-15	PD152	*Staphylococcus capitis*	1.975
Day-15	PD153	*Pseudomonas fragi*	2.020
Day-15	PD154	*Pseudomonas fragi*	1.944
Day-15	PD155	*Lactobacillus* sp.	1.887
Day-15	PD156	*Lactobacillus* sp.	1.769
Day-15	PD157	*Serratia proteamaculans*	2.408
Day-15	M151	*Serratia proteamaculans*	2.282
Day-15	M152	*Latilactobacillus sakei*	1.859
Day-15	M153	*Lactobacillus fuchuensis*	1.942
Day-15	M154	*Lactobacillus fuchuensis*	2.175
Day-15	M155	*Candida zeylanoides*	2.199
Day-15	M156	*Latilactobacillus curvatus*	2.149
Day-15	M157	*Latilactobacillus sakei*	2.500
Day-15	M158	*Latilactobacillus curvatus*	2.101
Day-15	M159	*Latilactobacillus curvatus*	1.985
Day-15	M1510	*Latilactobacillus curvatus*	2.268
Day-15	M1511	*Latilactobacillus curvatus*	2.321
Day-15	M1512	*Latilactobacillus curvatus*	2.035
Day-15	M1513	*Latilactobacillus sakei*	2.422
Day-15	M1514	*Latilactobacillus sakei*	2.439
Day-15	M1515	*Latilactobacillus curvatus*	2.193
Day-15	M1516	*Latilactobacillus curvatus*	2.140
Day-15	M1517	*Latilactobacillus sakei*	2.311
Day-15	M1518	*Latilactobacillus curvatus*	2.188
Day-15	M1519	*Latilactobacillus sakei*	2.429
Day-15	M1520	*Latilactobacillus sakei*	2.181
Day-15	M1521	*Latilactobacillus sakei*	2.532
Day-15	M1522	*Candida zeylanoides*	2.053
Day-15	M1523	*Latilactobacillus curvatus*	1.879
Day-15	M1524	*Candida zeylanoides*	2.330
Day-15	M1525	*Latilactobacillus sakei*	2.525
Day-15	M1526	*Latilactobacillus sakei*	2.383
Day-15	M1527	*Latilactobacillus curvatus*	2.064
Day-15	M1528	*Debaryomyces hansenii*	1.837
Day-15	M1529	*Latilactobacillus curvatus*	2.142
Day-15	M1530	*Latilactobacillus curvatus*	2.167
Day-15	M1531	*Latilactobacillus curvatus*	2.052
Day-15	M1532	*Latilactobacillus curvatus*	2.221
Day-15	M1533	*Latilactobacillus curvatus*	1.962
Day-15	M1534	*Lactobacillus fuchuensis*	1.829
Day-15	M1535	*Lactobacillus fuchuensis*	2.112
Day-15	M1536	*Candida zeylanoides*	2.158

For each sampling day and medium, up to a maximum of 70 colonies were selected for MALDI-TOF MS identification; when colony numbers exceeded this threshold, additional colonies were not included. Accordingly, the number of isolates varies by day and medium. Identification scores ≥2.00 were considered reliable for species-level identification and 1.70–1.99 for genus-level identification.

Supplemantary Table S2. Sensory descriptors, definitions and rating scale used in the evaluation of vacuum-packaged chicken breast meat, adapted from Katiyo et al. (2020) [8] for the five-point structured scale applied in the present study.

Attribute	Definition	1	2	3	4	5
**Odour**	The general odor perceived upon opening the packaging ranges in intensity from a neutral odor characteristic of fresh chicken to sour, sulfuric, ammonia-like, or putrid odors.	Fresh, neutral: no off-odour detected	Very slight sourness or vacuum odour: acceptable	Evident sour, sulphuric or off-odour; clear spoilage signal	Strong sour/putrid: ammonia or sulphur note: unacceptable	Very intense putrid: unconditionally rejected
**Appearance / Colour**	Surface colour, brightness and homogeneity; progressing from pink-cream fresh appearance toward grey, brown or greenish discolouration	Bright pink-cream: homogeneous, typical fresh appearance	Slight fading: minor colour heterogeneity	Evident paleness or shift to grey tone: dull surface	Brown-grey discolouration: onset of greenish patches	Complete discoloration, dark grey/green.
**Surface Texture / Slime**	Microbially derived slippery, sticky layer formation on the surface	Dry-moist, natural surface texture: no stickiness	Very slight increase in surface moisture: no stickiness	Noticeable stickiness, slightly sticky to the finger.	Evident slime formation: surface slippery	The slime is dense, with a surface completely covered in a mucous layer.
**Overall Acceptability**	Holistic quality judgement given after assessing all individual attributes	Completely fresh: no hesitation to consume	Acceptable: minor reservation	Borderline, hesitant, nearing the end of shelf life.	Unacceptable: would not consume	Completely spoiled: unconditionally rejected

Sensory characteristic definitions and evaluation framework adapted to chicken breast meat and five-point structured scale conditions, referencing Katiyo et al. (2020) [8]. The rejection threshold was assessed as an overall acceptability panel mean ≥ 3.0 or any of the odor or appearance scores ≥ 3.
